# 3D volumetric tomography of clouds using machine learning for climate analysis

**DOI:** 10.1038/s41598-025-90169-y

**Published:** 2025-03-10

**Authors:** Roi Ronen, Ilan Koren, Aviad Levis, Eshkol Eytan, Vadim Holodovsky, Yoav Y. Schechner

**Affiliations:** 1 Viterbi Faculty of Electrical & Computer Engineering, Technion - Israel Institute of Technology, Technion City, 3200003 Haifa, Israel; 2 Department of Earth & Planetary Sciences, Weizmann Institute of Science, Herzl St 234, 7610001 Rehovot, Israel; 3 Department of Computer Science, University of Toronto, Toronto, M5S 2E4 Canada; 4 Chemical Sciences Laboratory, National Oceanic and Atmospheric Administration, 325 Broadway, Boulder, 80305 CO USA; 5 Cooperative Institute for Research in Environmental Sciences, University of Colorado Boulder, 1665 Central Campus Mall, Boulder, CO 80309 USA; 6 David A. Dunlap Department of Astronomy & Astrophysics, University of Toronto, M5S 3H4, Toronto, Canada

**Keywords:** Inverse problems, Physics-based learning, Computer vision, Engineering, Scientific data

## Abstract

The prediction of climate has been a long-standing problem in contemporary science. One of the reasons stems from a gap in the ability to obtain 3D mapping of clouds, especially shallow scattered clouds. These clouds are strongly affected by mixing processes with their surroundings, rendering their internal volumetric structure highly heterogeneous. These heterogeneous clouds modulate the incoming solar energy and the outgoing long-wave radiation, thereby having a crucial role in the climate system. However, their 3D internal mapping is a major challenge. Here, we combine machine learning and space engineering to enable, for the first time, 3D mapping of scatterers in clouds. We employ ten nano-satellites in formation to simultaneously view the same clouds per scene from different angles and recover the 3D internal structure of shallow scattered clouds, from which we derive statistics, including uncertainty. We demonstrate this on real-world data. The results provide key features for predicting precipitation and renewable energy.

## Introduction

Clouds play a key role in the climate system by modulating incoming and outgoing radiation energy^1^. Specifically, shallow clouds are considered important coolers of the climate system. So, one of the key questions is the feedback of these clouds on global warming. Positive feedback would reduce cloud cooling^2^ and increase greenhouse warming.

**Fig. 1:**
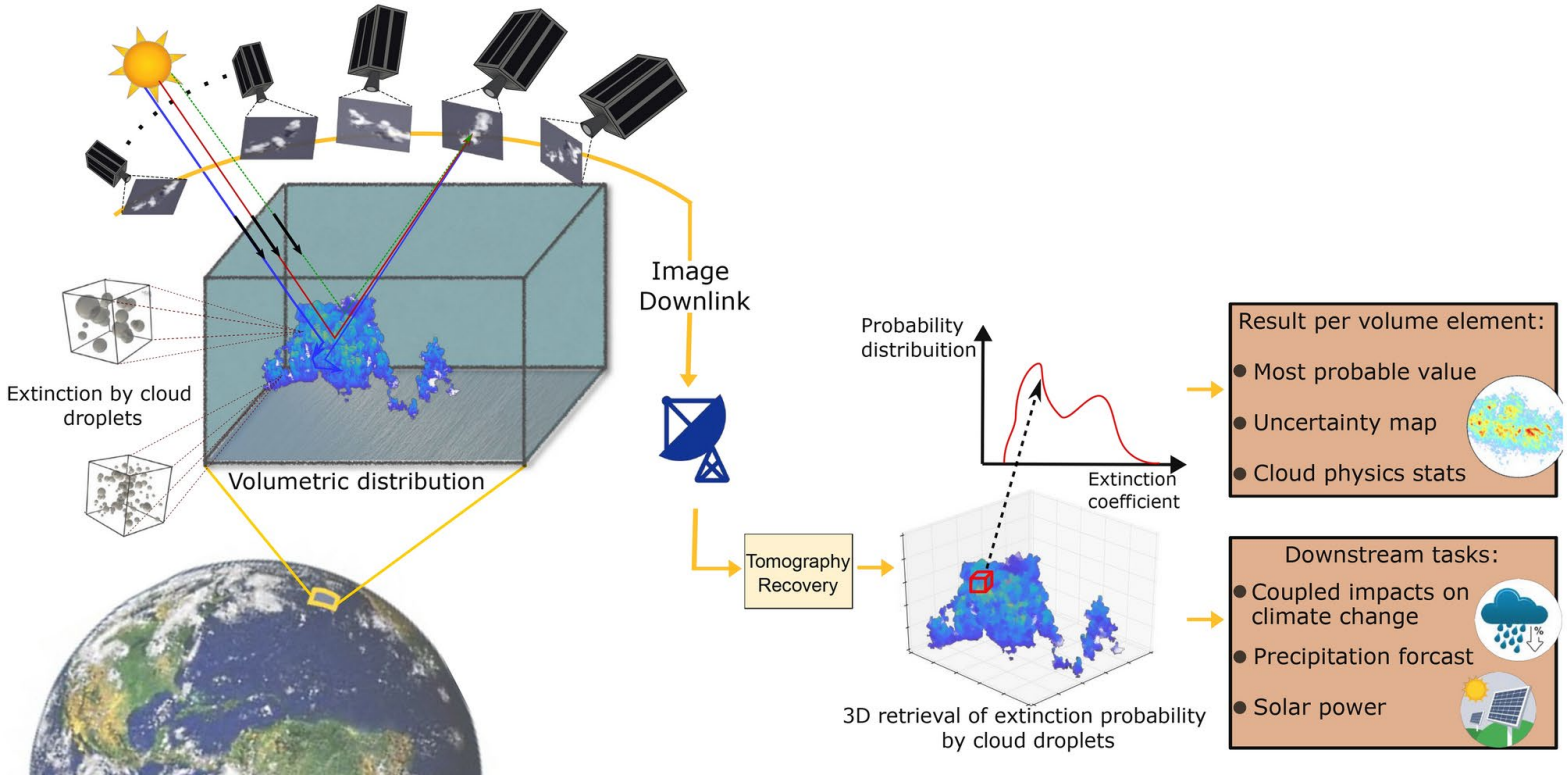
Spaceborne cloud tomography. In CloudCT, a coordinated satellite formation acquires images. Our learning-based tomography model processes the images. The model infers the posterior probability distribution of the cloud extinction coefficient at any point in a 3D domain. It thus infers a volumetric map of probability distributions (a distribution per location). The probability distribution yields multiple products, such as 3D maps of the cloud extinction coefficient’s most probable value and uncertainty, precipitation forecast, or ground-level solar power. The globe image was taken from the source pixabay.com/photos/earth-globe-planet-world-space-11015.

However, clouds, particularly shallow, sparse convective clouds, pose one of the largest challenges^2,3^ to climate models and prediction. There are several reasons for this. First, clouds are complex dynamical systems. They have feedback interactions between thermodynamic, microphysical, and radiative processes, all coupled in the scale range of $10^{-6} - 10^6 \text{m}$. Second, due to their small size, shallow, sparse clouds are more susceptible to mixing (including feedback) with the surrounding three-dimensional (3D) cloud-free environment^4^. Moreover, a typical grid element in a climate model is much larger than these clouds. Therefore, they are not resolved by the model but represented by sub-grid parameters that are supposed to capture their overall climate effect and sensitivities^5^. Therefore, shallow clouds are recognized as the primary source for climate prediction uncertainty^6^. The large variance in these predictions is attributed mainly to errors in cloud properties^7^.

To improve cloud representation in climate models, we need to understand better the *internal 3D structure* of small convective clouds. The dynamics and properties of these clouds have large variability and sensitivity to environmental conditions. Therefore, we need a way to volumetrically *measure* these clouds in a variety of conditions. Such measurements can help bridge a climate knowledge gap but require new approaches for spaceborne observations. Such rich observations and the *uncertainty* are important not only for climate but also for solar energy production and short-term weather forecasting through data assimilation^8,9^. Short-term forecasting has a significant societal importance. Weather prediction and intervention is affected by 3D cloud heterogeneity and processes, mainly regarding precipitation^10^ and cloud seeding by aerosols.

Retrieval of cloud properties by operational satellites simplifies structural complexity by assuming a *plane parallel* atmosphere^11^. Such an assumption implies cloud horizontal homogeneity over a large area: wide enough for cloud droplets’ interactions with solar radiation to degenerate to a 1D (vertical) effect. The plane parallel assumption breaks when dealing with small clouds. Their level of inhomogeneity does not permit a 1D assumption. Therefore, operational cloud products do not capture their properties^12,13^.

Radars yield 3D content in clouds but are not fully compatible with this need. Weather radars are excellently sensitive to precipitating particles (e.g., raindrops) of mm scale^14–16^. Shallow-cloud droplets are about two orders of magnitude smaller. Radar reflectivity generally increases^17,18^ with the droplet radius to a power of six. Hence, weather radars are much less sensitive to characteristics of small cloud droplets^16^. This issue stems from the typical wavelengths of X-band ($\sim 3$cm) and W-band ($\sim 3$mm). In contrast, optical wavelengths are more comparable to cloud droplets, thus offering much better sensitivity in this regime. Moreover, due to their wavelengths, radars typically have much coarser spatial resolution^19^: the state-of-the-art EarthCARE radar^17^ has native vertical resolution and along-track resolution of 500m, using a 2.5m antenna. This resolution is comparable to the overall size of a shallow cumulus cloud. Furthermore, using limited resources, the need to actively project electromagnetic power limits the spatial coverage or temporal sampling of radars and lidars^20^.

This paper takes an entirely different and unprecedented way (see Fig. 1). For the first time, we present the *reconstruction of the 3D internal structure* of shallow scattered clouds, extracting statistics and uncertainty. We employ 10 nano-satellites and machine learning (ML) to do that. We demonstrate this on real-world NASA data and intensive simulations. Our volumetric retrieval is a unique form of computed tomography (CT) based on multi-view spaceborne optical images. The results critically report *uncertainty* by design. To demonstrate this, we derive the CloudCT space mission^21,22^, funded by the ERC. Spaceborne multi-view *simultaneous* imaging of a field of small clouds in *decameter* resolution requires multiple satellites that fly in a coordinated *formation*. This capability is feasible thanks to the advent of nano-satellites, whose low cost facilitates them being built and placed in large numbers.

This novel type of CT cannot rely on the mathematical heritage of medical imaging, which assumes a linear radiative transfer (RT) model. Wide field cloud imaging is passive, relying solely on solar radiation *multiply scattered* by droplets and other scene components. Scattering *creates* the signal. Raw image data relate *nonlinearly* to 3D volumetric cloud structure by 3D RT. This concept brings new challenges: (a) Numerically, 3D RT is a nonlinear recursive operation. It is computationally very difficult to invert. For large scale, it is impractical to use physics-based differential rendering in an iterative optimization approach^23–27^, for 3D scattering-based CT. (b) Nonlinearity challenges computation of *uncertainty*. (c) Contrary to controlled imaging settings, as in microscopy and medical CT, spaceborne imaging has variable geometry due to the motion of the cooperating platforms.

The concept of cloud scattering-CT, described above, is new. It addresses the critical challenges for the first time. In conjunction with the concept of satellite formation for data acquisition, ML is key to meeting the analysis challenges. ML shifts the computational burden to a *training stage*. Consequently, at inference, extensive data can be scalably analyzed at rates expected from spaceborne downlink. Moreover, training on clouds implicitly encodes their structural nature in a model. The encoding thus serves as a prior during cloud inference.

Therefore, as part of the CloudCT concept, we derive an ML model for data analysis, termed ProbCT. As described in the Methods section, the inputs of ProbCT comprise multi-view image data, 3D camera locations, and the 3D coordinates of a queried location in the atmospheric domain. Then, per 3D location in the medium, ProbCT estimates a *function*: the posterior probability distribution of the extinction coefficient. The inferred probability distribution enables the extraction of statistics. These include per-location, the maximum a-posteriori (MAP) estimate, an expected value, and uncertainty measures.

Training requires a large set of labeled ground-truth clouds whose volumetric contents are known in 3D. Ideally, this would rely on hundreds of thousands of in-situ simultaneous measurements per cloud. However, it is not feasible to empirically obtain such extensive in-situ data in nature, either for training or testing. Thus, our approach uses simulations created and validated by the cloud physics community. These are state-of-the-art, high spatiotemporal resolution, bin-microphysics cloud-field simulations. They integrate thermodynamics, multi-phase fluid dynamics, and stochastic processes. We incorporate a variety of empirical environmental boundary conditions, resulting in different cloud classes, each class having its own statistics. Each cloud scene yields corresponding image data using 3D RT, including noise. We thus provide the first large database of multi-class volumetric cloud fields and their corresponding images.

Such supervised training has a potential vulnerability: real observations may have clouds whose class is poorly sampled or absent from the training set. So, inspired by physics-based learning^28^, training is augmented by self-supervised learning, that uses real-world cloud images.

We validate the concept of cloud tomography in such extensive simulations and demonstrate it using data from NASA’s AirMSPI^29^. AirMSPI is mounted on NASA’s ER-2 and acquires multi-angular sequential images of cloud fields. This work marks significant advances relative to prior work on scattering-based CT^23–27,30^. The prior art sought only a single value per voxel variable, while ProbCT estimates a posterior distribution function. This major and novel generalization yields uncertainty measures critical for scientific and operational conclusions. The principle of ML as a mechanism for solution is much more robust than prior art^30^ due to training and stress testing across various cloud classes (rather than a single class) and the introduction of self-supervision. Self-supervision enables ProbCT, unlike the prior art, to adapt to cloud types that are out of the training class, alleviating a barrier to real use. Finally, we demonstrate downstream uses for renewable energy, precipitation forecasting, and remote sensing of the adiabatic fraction.

## Cloud tomography results

First, we use an imaging geometry as in CloudCT. It has 10 satellites, having 100km between nearest neighboring, perspective viewpoints, orbiting 500km high, using wavelength around 670 nm.

The 3D fields of true and estimated cloud extinction coefficients are denoted ${\varvec{\beta }}^\text{true}$ and $\hat{\varvec{\beta }}$, respectively.

**Fig. 2:**
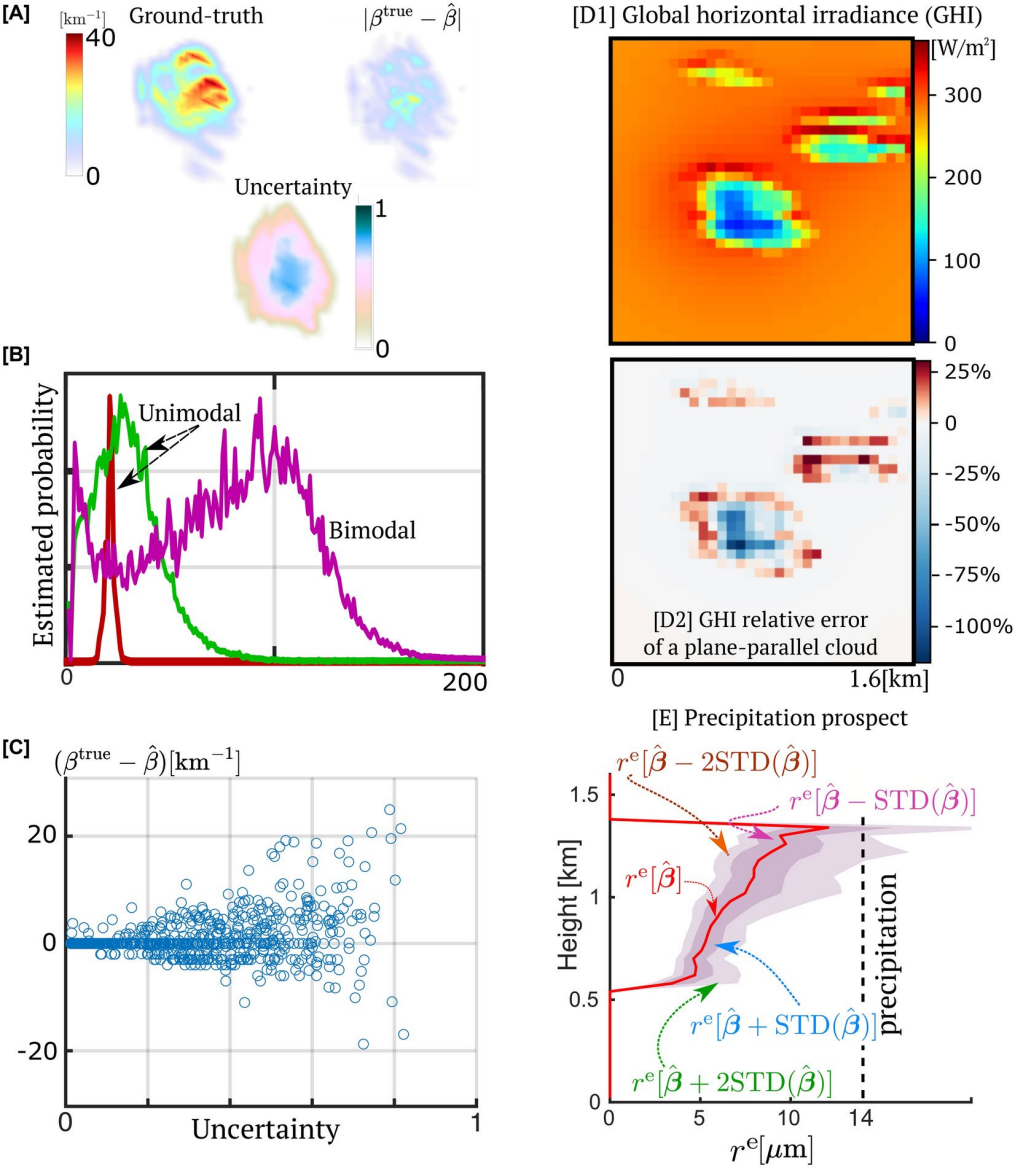
Simulated results. (**A**) Visualizations of 3D volumetric fields by MIP at side-view: A labeled test cloud, its estimation error, and uncertainty (normalized entropy). They increase at the cloud core. (**B**) Sample inferred probability distributions normalized by the MAP value. (**C**) Inferred results at 2000 voxels, which were randomly sampled across the test set. A high inferred normalized entropy (uncertainty) implies a possible large absolute error. Large errors of ${\hat{\beta }}$ rarely occur when the inferred entropy is low. (**D1**) The global horizontal irradiance (GHI) on the ground, under a partly cloudy sky^31^. (**D2**) The relative error (Eq. 13) is caused by an erroneous assumption of horizontally uniform clouds. (**E**) Uncertainty in ${\hat{\varvec{\beta }}}$ propagates to estimation of the cloud droplet effective radius $r^\text{e}$. A value $r^\text{e}>14\mu \text{m}$ is a *precipitation trigger*^32^, yielding rain and dramatic shortening of cloud life.

Figure 2A uses maximum intensity projection (MIP) to visualize a cloud, the recovery error $|\hat{\varvec{\beta }}- {\varvec{\beta }}^\text{true}|$ and uncertainty. Noisy multi-view image data is denoted $\mathbf{y}$. Figure 2B plots example probability distribution functions ${\hat{P}}(\beta |\mathbf{y})$, that ProbCT infers for some locations in the cloud. Per location, we estimate the extinction coefficient $\hat{\beta }$ and the uncertainty by MAP and the normalized entropy of the inferred probability distribution function, respectively. The error and uncertainty increase mainly in the cloud core, in agreement with theory^33^: light undergoes many scattering events until it reaches the core and afterward on the way to the cameras. Imaging noise then overwhelms the core’s signal.

Randomly sampling voxels from all test clouds, the estimation errors are scatter-plotted vs. uncertainty in Fig. 2C. Large errors occur only where the inferred normalized entropy is high. That is, ProbCT indicates where its estimation of the extinction coefficient $\beta$ may fail.

**Fig. 3:**
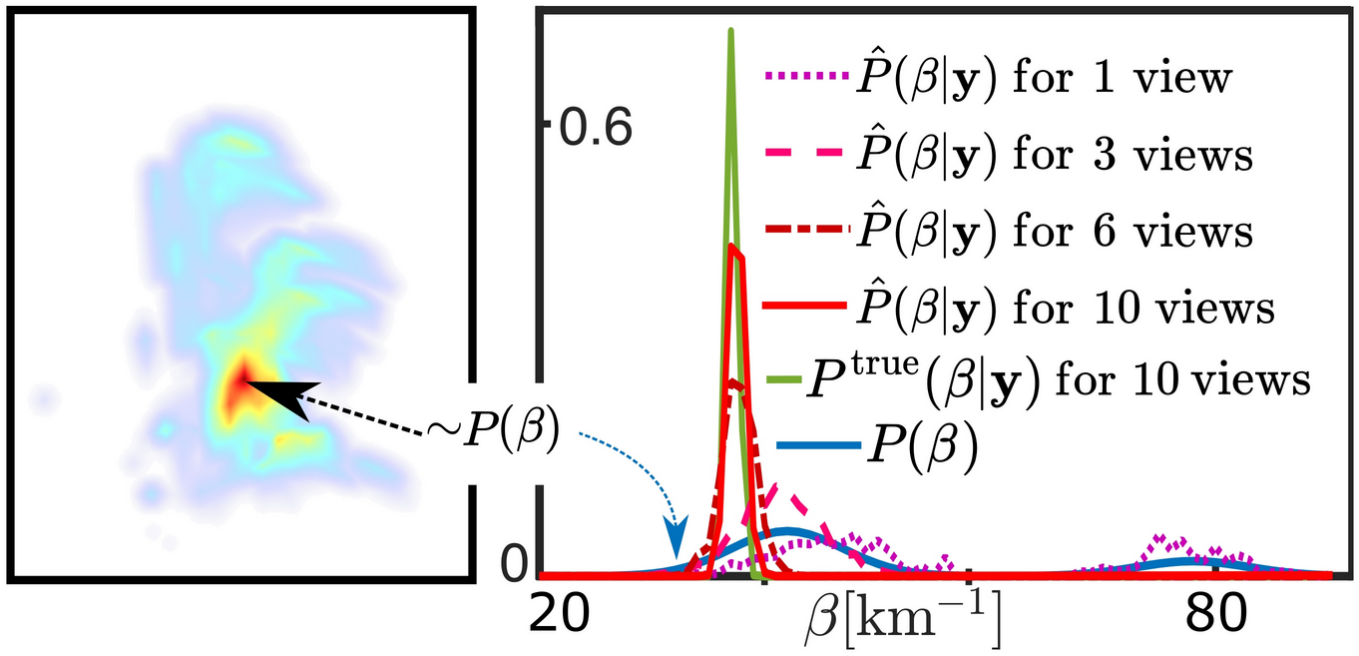
Necessity of multi-view data for cloud tomography. [Left] Clouds differ by a single voxel (pointed out by a black arrow). Here is a visualization of ${\varvec{\beta }}$ by MIP at $45^\circ$ off-nadir for a single test cloud. [Right] Blue: the prior probability distribution from which $\beta$ at the voxel is drawn. Green: the sharply peaked true posterior of $\beta$ in this voxel of a test cloud by 10 views. Other lines plot inferences of the posterior probability distribution for different numbers of views. As this number increases, ${\hat{P}}(\beta |\mathbf{y})\rightarrow P^\text{true}(\beta |\mathbf{y})$, which is sharply peaked around the true value.

The success of the approach is not only due to training. Rather, it stems from information carried by multi-view geometry, essential for CT. To show this, we vary the number of viewpoints. The training and testing datasets here are made of clouds that differ by a single voxel in the cloud core. In this voxel, $\beta$ is sampled randomly from a bimodal probability distribution $P(\beta )$. For this case, the actual a-posteriori probability distribution function $P^\text{true}(\beta |\mathbf{y})$ is derived in the supplementary information. Figure 3 presents the results. From a single viewpoint, data is insufficient for CT recovery despite training. Thus, the inferred posterior probability follows the true one, which in this case is simply the prior of $\beta$, that is ${\hat{P}}(\beta |\mathbf{y})\rightarrow P^\text{true}(\beta |\mathbf{y})\sim P(\beta )$. Hence, estimation is oblivious to the image data and is as random as the set of clouds. On the other hand, as the number of viewpoints increases, the estimated posterior reaches the true analytic posterior, ${\hat{P}}(\beta |\mathbf{y})\rightarrow P^\text{true}(\beta |\mathbf{y})$, which is sharply peaked at ${\beta }^\text{true}$.

Figure 2D,E illustrate applications to renewable energy and precipitation. The atmosphere (including clouds and air molecules) affects solar radiation reaching the ground non-linearly in opposite ways: the atmosphere attenuates direct solar irradiance, while diffuse irradiance typically increases with atmospheric scatterer density. Figure 2D1 shows a two-dimensional map of the expected global horizontal irradiance (GHI) $[\frac{\text{W}}{\text{m}^{2}} ]$ on the ground, derived from an estimated cloud field. The GHI and its uncertainty are important for climate and weather predictions and renewable electric power generation from solar energy. The GHI can have significant errors if it is based on an assumption of horizontal homogeneity in a cloud (rather than 3D heterogeneity). This is illustrated in Fig. 2D2, which correspondingly maps the relative error due to such an assumption.

A threshold size of cloud droplets triggers precipitation^32^. Per voxel, the effective radius of the droplets can be estimated via $\hat{\beta }$. Let ${r}^\text{e}[{\varvec{\beta }}]$ be the effective radius averaged horizontally at the cloud core. The uncertainty in ${\hat{\varvec{\beta }}}$ propagates to corresponding plots of ${r}^\text{e}$. Figure 2E plots ${r}^\text{e}[\hat{\varvec{\beta }}]$ as a function of altitude. It indicates that the cloud likely does not precipitate, but there is a slight chance otherwise.

**Fig. 4:**
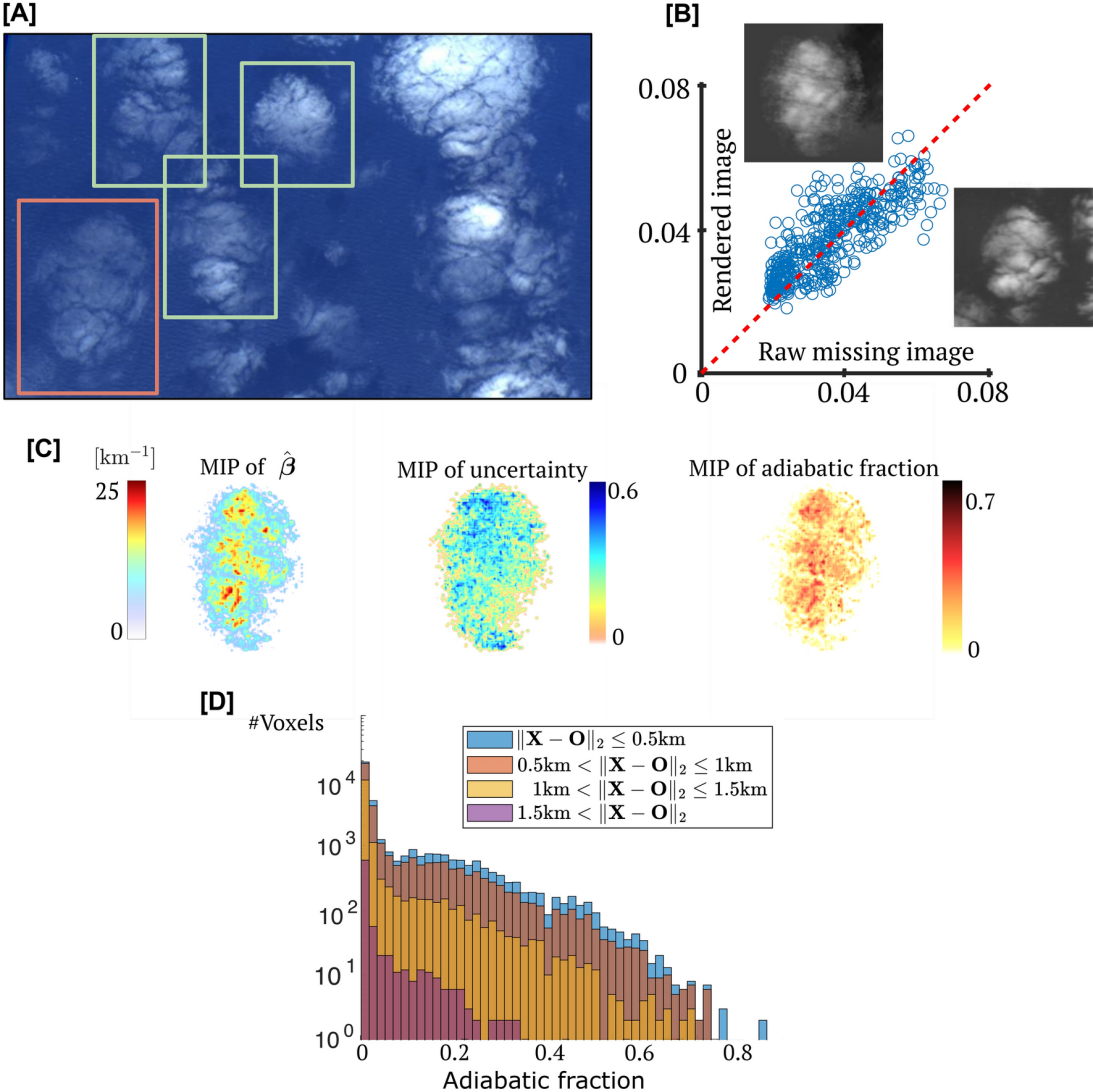
Real-world experiment. (**A**) Nadir image from a NASA AirMSPI flight^29^ at 20:27GMT on February 6, 2010 over 32N 123W. Green rectangles: regions used for self-supervised training. Red rectangle: a test domain. (**B**) Comparing (visually and by a scatter plot) an AirMSPI image excluded from inference vs. an image rendered in the corresponding viewpoint based on the inferred cloud. For clarity, the scatter plot uses $1\%$ of the image pixels. (**C**) MIP of the inferred MAP ${{\hat{\beta }}}$ of the cloud, MIP of the uncertainty (normalized entropy), and MIP of an estimated adiabatic fraction. (**D**) Histogram of the estimated adiabatic fraction. Bar colors represent voxel distance from the cloud center $\mathbf{O}$. As expected, the adiabatic fraction decreases as the distance from the cloud core increases.

We use real-world images to demonstrate CT of a real-world cloud imaged by NASA’s AirMSPI instrument^29^. AirMSPI takes nine pushbroom multi-angular images from 20 km altitude, in a $\pm 67^\circ$ span along-track, with 10 m ground resolution at nadir and a wavelength band around $660\text{nm}$. We inferred a volumetric domain (see Fig. 4), having $72\times 72 \times 32$ voxels, i.e., 165,888 unknowns.

There is no ground truth map of the extinction coefficient of a real-world cloud. Therefore, we check for consistency using cross-validation. For this, we excluded the $+47^\circ$ view from the input. Then, ProbCT inferred both ${\hat{\varvec{\beta }}}$ and the uncertainty field, using only eight viewpoints. Afterward, we used RT to render the missing view. Quantitatively, the root mean square error between the estimated cloud re-rendered image and the excluded image is $25\%$ of the highest value observed in the excluded image.

Figure 4C,D refers to a novel product based on 3D recovery. The liquid water content ($\text{LWC}$ in $[\mathrm{g/m}^3]$) in a volume element is affected by mixing and therefore varies in 3D. The theoretical baseline adiabatic case assumes no mixing. Then, correspondingly, $\mathrm{LWC^{ad}}$ is horizontally homogeneous and can be calculated analytically as a function of altitude above the cloud base^34^. So, given a cloud base, the ratio $\text{LWC}/\mathrm{LWC^{ad}}$ (termed *adiabatic fraction*) can be computed. It is an important measure of mixing and dilution. To our knowledge, this measure has not been experimentally retrieved in 3D by remote sensing. We present the first results in this direction, where $\text{LWC}$ is assessed using the inferred ${\hat{\beta }}$, per location.

Theory and measurements indicate that an undiluted *cloud core* lies in the cloud center, while near the cloud edge, there is a transition zone, which is 10-100m thick. In this zone, small-scale turbulent mixing with the environment is intense^35–38^. This zone contains regions having^35,39^ a low adiabatic fraction, i.e., smaller than 0.2. Figure 4C,D shows remarkable agreement with this former knowhow. The result indicates, for the first time, a proof of concept for retrieving the adiabatic fraction by remote sensing.

## Discussion

This work introduces passive scattering tomography of clouds and key technologies to enable this from space, as pursued by *CloudCT*. These include the use of a formation of satellites to acquire multi-view images of a cloud field simultaneously and tomographic analysis based on machine learning. The analysis system, titled *ProbCT*, yields for the first time, per 3D location, the posterior probability distribution function of the extinction coefficient, $P(\beta |\mathbf{y})$. Consequently, per location, it is possible to estimate an optimal value of the extinction coefficient and *uncertainty.* A distribution can yield other products, such as high-order moments, the number of modes (e.g., a mode of low extinction and a mode of high extinction), and their weight. Inference run-time by ProbCT is comparable to the downlink rate from orbit.

The uncertainty field provides interesting research prospects. In state-of-the-art cloud physics, weather, and climate predictions, significant knowledge gaps stem from the interaction between the multi-scale turbulent flow and microphysical processes. Much of the uncertainty in this interaction and thus in these scientific fields relate to cloud mixing with surrounding air and processes at the cloud margins^40–43^. The results here show that in these regions, retrieval by CT tends to have the lowest observation uncertainty, fortunately. This finding indicates that scattering-based CT is highly suited to help resolve some current scientific uncertainties. Our results also show that retrieval by CT tends to have high observation uncertainty at the cloud core. However, the cloud core has the least dilution with the air outside. The cloud core structure tends to follow simpler physics models, specifically the adiabatic model^34^. Thus, observational uncertainty at the core may have smaller consequences, though further study is needed to assess this.

Uncertainty quantification has practical use when using cloud data to tune physical models. For example, rain initiation in small clouds is a bifurcation point: the cloud can rain down to Earth’s surface or remain a little longer until it evaporates. Another example is the study and representation of the effects of aerosol and air pollution on clouds and climate^44,45^. Observations constrain researched models. Then, clouds with high uncertainty (based on ProbCT) can be dropped out or weighted down during data analysis and tuning of models.

There is a practical limit to inference based on training. Training is based on samples drawn from a distribution of either real or simulated environmental conditions. Moreover, simulated clouds may not express the full complexity of nature. It is difficult to guarantee that a distribution used during training matches the diversity of nature. Inconsistency of the distribution increases estimation errors and uncertainty. We attempt to quantify this aspect in this paper by inferring clouds drawn from several databases corresponding to different environmental conditions. Nevertheless, this limitation merits further study and mitigation.

The problem we tackle has fundamental limits due to the randomness of light and matter. Fundamentally, image data is noisy due to the Poissonian nature of photons. Then, inverse scattering is ill-posed, regardless of the estimator: various multiply-scattering volumetric contents can “explain” the measured noisy data. Hence, even the true $P(\beta |\mathbf{y})$ is *not* a delta function, which has zero variance around a single possible $\beta$. Thus, any good estimator (ProbCT as well) is not expected to generally output a delta function for ${{\hat{P}}}(\beta |\mathbf{y})$. This limitation can be partly addressed if data includes additional, independent sources (though they are also noisy). These may be environmental temperature and humidity profiles sampled globally by various meteorological instruments^46^ or data sources mentioned below. Moreover, there is high randomness in the *nature* of object matter here. The reason is that *chaotic, turbulent* flow drives the creation of convective clouds. Thus, inference may ultimately have a limit to generalizing from prior turbulent fields to represent a novel field.

There are several ways to extend this work. One is to recover the joint distribution of several parameters per location (single-scattering albedo, cloud droplet sizes, and their density). Another important extension is incorporating the variability of solar paths and diversity of land cover. The latter can be drawn from validated data sources, such as SRTM DEM^47^ or LULC^48,49^. Additionally, it will be helpful to assimilate other sources in the encoding stage, not only regarding shallow clouds but potentially to lead to similar research into deep clouds. Various sources already used and assimilated in meteorology, such as models, radar, and long-wavelength imaging, may be used with the optical visible data. Radars excel at probing deep clouds, raindrops, and hail, complementing optical sensing. Radars also retrieve velocities by Doppler shifts. These additions would enhance the realism of the framework and can be done both in supervised and self-supervised training. Such improvements can better assist downstream applications such as renewable energy and weather forecasting.

This work opens the door to new ways of scientific analysis relating to remote sensing and atmospheric physics. It indicates that complex multi-view images can realistically be acquired and processed to shed light on hard questions involving 3D heterogeneity and multiple scattering. Moreover, some of our principles may be relevant to additional domains where multiple scattering and/or reflections are significant, such as medical imaging, non-line-of-sight imaging, and reflectometry.

## Methods

The input for analysis by ProbCT is a set of multi-view images of a field of shallow clouds and the related camera (satellite) locations. To train the ProbCT ML system, we provide a large number of sets, *N*, for which the actual extinction 3D field is fully known in advance and thus labeled. We use state-of-the-art, high-resolution, cloud-resolving models to obtain a labeled set. The output of these cloud-resolving models is a 3D volumetric description of a time-evolving cloud field, including its microphysics.

Such an output simulated cloud field serves as labeled input for a forward RT solver. The RT solver calculates the radiation field in 3D when the sun illuminates the cloud scene. This field creates a set of calibrated expected images from various camera poses. The images are then randomly perturbed according to imaging noise models. The labeled cloud fields and their corresponding multi-view image sets serve as input for training ProbCT.

### Synthetic 3D cloud fields

The numerical cloud models should accurately describe the cloud field. Moreover, their outputs should span a wide enough variety of shallow cloud cases to capture the variety of their macro and micro properties. The simulated shallow cloud fields differ in their thermodynamic profiles and aerosol loading. For all of the marine cases, we have used the system for atmospheric modeling (SAM), which runs in a large eddy simulation (LES) mode and fully resolves boundary layer clouds in a non-hydrostatic, anelastic model^50^. The LES is coupled to a microphysical model that explicitly solves the governing process of droplet nucleation and growth (HUJI SBM^51^).

The atmospheric profiles were measured by radiosondes for cases of shallow scattered clouds both over the ocean and overland^35,46,52,53^. Aerosol properties affect droplet activation and derive dynamical feedback that determines the overall cloud field properties^54^. Per each profile, we run several aerosol loading scenarios spanning highly pristine to highly polluted conditions. Details about the parameters of the simulations, as well as the database size, are listed in the supplementary information.

### Multi-view cloud rendering

Consider a 3D volumetric field of the cloud extinction coefficient, ${\varvec{\beta }}$. Besides cloud droplets, air molecules affect RT. Throughout this paper, we model the molecular extinction coefficient ${\varvec{\beta }}^\text{air}$ using a summer mid-latitude vertical distribution^24^, at altitudes in the range [0, 20]km. The medium is also characterized by a single-scattering albedo and a scattering phase function. Their values stem from generated microphysical properties of the mixture^55^ of particles in a voxel, including air and water droplets. For self-supervised training and for rendering AirMSPI results, we follow^24,30,55,56^, using a 10$\mu \text{m}$ droplet effective radius and effective variance of 0.1. The *forward model* ${{\mathscr {F}}} \left( {\varvec{\beta }} \right)$ constitutes 3D RT followed by projection to all cameras and consequent sampling to pixels. This paper uses the SHDOM^57,58^ RT solver. The supplementary information provides additional details on the 3D RT equations and their numerical modeling.

A vector represents the acquired multi-view image data

1 $$\begin{aligned} \textbf{y}= {{\mathscr {N}}} \left\{ {{\mathscr {F}}} \left( {\varvec{\beta }} \right) \right\} \;\;. \end{aligned}$$

Here, the operator ${{\mathscr {N}}}$ introduces random imaging noise. The noise in AirMSPI training and real data complies with specifications described in^29^. For forming perspective cameras (as in CloudCT), we use noise specifications derived from the CMV4000 sensor, having a pixel size of $5.5 \times 5.5$ $\mu m^2$. The exposure time adjusts to the radiance that reaches the camera: the maximum image-pixel value corresponds to 90% of the sensor full well, which is 13,500 photo-electrons. Thus, sampled radiance is converted to a Poissonian distributed photo-electron count. There are 13 photo-electrons per gray level. The readout noise STD is 13 electrons. The camera uses 10-bit quantization.

### ProbCT ML model

Per queried 3D location $\mathbf{X}$ in the atmospheric domain, ProbCT infers the posterior probability distribution function, ${{\hat{P}}}_{\mathbf{X}}(\beta |\mathbf{y})$. During inference, the inputs comprise image data denoted $\mathbf{y}$, acquired from $N^\text{cam}$ viewpoints, each indexed *c* and corresponding 3D camera locations $\{\mathbf{X}_{c} \}_{c=1}^{N^\text{cam}}$. The input also includes $\mathbf{X}$, which is queried if it passes a space carving cloud mask, based on the multi-view images^24^. The ProbCT architecture comprises an encoder and a decoder, controlled, respectively, by sets of learned parameters, ${\varvec{\Theta }}^\text{enc}$ and ${\varvec{\Theta }}^\text{dec}$. Overall, the set of system parameters is

2 $$\begin{aligned} {\varvec{\Theta }}=[{\varvec{\Theta }}^\text{enc}, {\varvec{\Theta }}^\text{dec}] \;. \end{aligned}$$

Per $\mathbf{X}$, the encoder outputs a vector $\mathbf{u}_\mathbf{X}({\varvec{\Theta }}^\text{enc})$, whose dimensions are much larger than the combined dimensions of voxel and camera poses and the number of image pixels that relate to $\mathbf{X}$. A decoder then acts on $\mathbf{u}_\mathbf{X}$, *decreasing* dimensions down to a short, discrete representation (vector) of the estimated posterior probability ${{\hat{P}}}_{\mathbf{X}}(\beta |\mathbf{y}, {\varvec{\Theta }})$ at $\mathbf{X}$, of length *Q*. It corresponds to quantized values $\beta (q)=q\Delta \beta$, where $q\in [0,\ldots ,Q-1]$ and $\Delta \beta$ is a quantization step. This discretization occurs only during inference, not while rendering a true cloud or during error quantification. The supplementary information details the encoder and decoder architectures.

Throughout the paper, we estimate $\beta$ at $\mathbf{X}$ using the MAP criterion, that is, the extinction bin with the highest estimated posterior probability:

3 $$\begin{aligned} {{\hat{\beta }}}(\mathbf{X})= \Delta \beta \cdot \arg \! \max _q {{\hat{P}}}_{\mathbf{X}}(q\Delta \beta |\mathbf{y}, {{\varvec{\Theta }}}). \end{aligned}$$

Using Eq. (3) $\forall \mathbf{X}$ estimates the 3D volumetric object. Uncertainty can be quantified by entropy. Uncertainty is maximal when all potential values of $\beta$ have equal probability, in which case the entropy is ${\log _2 Q}$. We use the normalized entropy

4 $$\begin{aligned} H_\mathbf{X}^\text{norm} = \frac{-\sum _{q=0}^{Q-1} \left[ {{\hat{P}}}_\mathbf{X}(q\Delta \beta |\mathbf{y},{{\varvec{\Theta }}}) \log _2 {{\hat{P}}}_\mathbf{X}(q\Delta \beta |\mathbf{y},{ {\varvec{\Theta }}}) \right] }{\log _2 Q} , \end{aligned}$$

for which $0\le H_\mathbf{X}^\text{norm}\le 1$. Here $H_\mathbf{X}^\text{norm}=0$ for absolute certainty, where ${{\hat{P}}}_\mathbf{X}(q\Delta \beta |\mathbf{y},{{\varvec{\Theta }}})$ is a delta function. On the other hand, $H_\mathbf{X}^\text{norm}=1$ when ${{\hat{P}}}_\mathbf{X}(q\Delta \beta |\mathbf{y},{{\varvec{\Theta }}})$ is uniformly distributed, corresponding to maximum uncertainty.

#### Supervised training

 A set of *N* labeled pairs $\{ ({\varvec{\beta }}^\text{true}_n,\mathbf{y}_n) \}_{n=1}^N$ is used for supervised training of ${\varvec{\Theta }}$. Per labeled cloud datum, the true probability distribution at $\mathbf{X}$ is discretized and represented by a one-hot vector, whose $q^\text{th}$ element equals 1,

5 $$\begin{aligned} P_{\mathbf{X}}^\text{true}(q\Delta \beta )= {\left\{ \begin{array}{ll} 1 & \text {if}~~~ q=\lfloor \beta ^\text{true}(\mathbf{X})/\Delta \beta \rfloor \\ 0 & \text {otherwise} \end{array}\right. }\;. \end{aligned}$$

On the other hand, ProbCT infers a corresponding vector ${{\hat{P}}}_{\mathbf{X}}(q\Delta \beta |\mathbf{y},{\varvec{\Theta }})$. Training seeks to minimize the distance between these discrete probability distributions. This distance is generally quantified by the Kullback-Leibler divergence^59^. Optimizing this divergence, in our case, is equivalent to minimizing the cross-entropy between these distributions, defined by

6 $$\begin{aligned} \text{CE}_\mathbf{X}(\mathbf{y},{\varvec{\Theta }})= \!-\! \sum _{q}\! \left[ P_{\mathbf X}^\text{true}(q\Delta \beta ) \log {{\hat{P}}}_\mathbf{X}(q\Delta \beta |\mathbf{y},{\varvec{\Theta }}) \right] = -\log {{\hat{P}}}_{\mathbf{X}} \left( \left. \left\lfloor \frac{\beta ^\text{true}(\mathbf{X})}{\Delta \beta } \right\rfloor \Delta \beta \right| \mathbf{y},{\varvec{\Theta }} \right) .~~ \end{aligned}$$

Aggregating Eq. (6) over all voxels and labeled scenes, supervised training solves this optimization form

7 $$\begin{aligned} ( {\hat{\varvec{\Theta }}}^\text{enc}_\text{super}, {\hat{\varvec{\Theta }}}^\text{dec}_\text{super} ) = \arg \!\min _{\varvec{\Theta }} \sum _{n=1}^N \sum _{\mathbf{X}} w^\text{cloud}_{\mathbf{X},n} \text{CE}_\mathbf{X} (\mathbf{y}_n, {\varvec{\Theta }}) . \end{aligned}$$

Here $w^\text{cloud}_{\mathbf{X},n}$ is a weight. Most voxels in a scene are empty, having $\beta =0$. A small minority of voxels are in a cloud ($\beta >0$). We found in practice that if the contribution of empty voxels to the optimization loss (Eq. 7) is not weighted down, the system trains to focus too much on void areas. In all cases, $w^\text{cloud}_{\mathbf{X},n}=1$ if in scene *n*, $\beta ^\text{true}(\mathbf{X})\ge \Delta \beta /2$, that is, $\mathbf{X}$ is a cloud voxel. Empty voxels ($\beta ^\text{true}(\mathbf{X})<\Delta \beta /2$) assign a smaller weight.

#### Self-supervised training

 Define a set of unlabeled data $\{ \mathbf{y}_m\}_{m=1}^M$ of *M* imaged clouds. During self-supervised training, rendering ${{\mathscr {F}}}$ assumes a fixed default cloud phase function and albedo. Using ${\varvec{\Theta }}^\text{enc}, {\varvec{\Theta }}^\text{dec}$, running Eq. (3) $\forall \mathbf{X}$ estimates a 3D volumetric object denoted ${\hat{\varvec{\beta }}}_m(\mathbf{y}_m, {{\varvec{\Theta }}}^\text{enc}, {{\varvec{\Theta }}}^\text{dec})$. The forward model ${{\mathscr {F}}}$ of 3D RT renders images of ${\hat{\varvec{\beta }}}_m.$ Define a cost by the squared difference between true image pixel values and the corresponding pixel values of the re-rendered estimated cloud,

8 $$\begin{aligned} E({\varvec{\Theta }}^\text{enc}, {\varvec{\Theta }}^\text{dec}) = \sum _{m=1}^M \left\| \mathbf{y}_m - {{\mathscr {F}}} \left\{ {\hat{\varvec{\beta }}}_m (\mathbf{y}_m, {\varvec{\Theta }}^\text{enc}, {\varvec{\Theta }}^\text{dec}) \right\} \right\| _2^2. \end{aligned}$$

Then, minimizing *E* yields learning. This minimization leverages stochastic gradient descent based on *differential rendering*. Differential rendering expresses how ${{\mathscr {F}}}$ changes due to small deviations in ${\hat{\varvec{\beta }}}_m$. In principle, the whole set of parameters $({\varvec{\Theta }}^\text{enc}, {\varvec{\Theta }}^\text{dec})$ can be optimized. However, we opted to keep the *encoder* parameters fixed at ${\hat{\varvec{\Theta }}}^\text{enc}_\text{super}$ (obtained by Eq. 7). Hence, we refine only ${\varvec{\Theta }}^\text{dec}$:

9 $$\begin{aligned} {\tilde{\varvec{\Theta }}}^\text{dec} = \arg \min _{{\varvec{\Theta }}^\text{dec}} E({\hat{\varvec{\Theta }}}^\text{enc}_\text{super}, {\varvec{\Theta }}^\text{dec}) \;. \end{aligned}$$

This optimization is initialized by ${\hat{\varvec{\Theta }}}^\text{dec}_\text{super}$ from Eq. (7).

All operations required for Eq. (9) are differentiable, except the $\arg \!\max$ operator in Eq. (3). We approximate this operator using a differential $\texttt {Smoothmax}$ (Boltzmann) operator^60^. Set a parameter $\alpha >0$. Define a probability distribution

10 $$\begin{aligned} { \Phi }_{\mathbf{X}}(q) = \frac{\left[ {{\hat{P}}}_{\mathbf{X}}(q\Delta \beta | \mathbf{y},{{\varvec{\Theta }}}) \right] ^\alpha }{ \sum _{q'} \left[ {{\hat{P}}}_{\mathbf{X}}(q'\Delta \beta | \mathbf{y}, { {\varvec{\Theta }}}) \right] ^\alpha } \;. \end{aligned}$$

It has a property that for$\alpha \rightarrow \infty$, ${ \Phi }_{\mathbf{X}}({{\hat{q}}}) \rightarrow \delta ({q-{{\hat{q}}}})$, where ${{\hat{q}}}$ is given in Eq. (3). Define${{\mathbf{b}}}={\Delta \beta }\cdot [0,1,\dots ,Q-1]$ and ${\varvec{\Phi }}_{\mathbf{X}}=[{ \Phi }_{\mathbf{X}}(0),{ \Phi }_{\mathbf{X}}(1),\dots ,{ \Phi }_{\mathbf{X}}(Q-1)]^\top$, where $\top$ denotes transposition. A differential approximation to Eq. (3), yielding a continuous value is

11 $$\begin{aligned} {{\hat{\beta }}}(\mathbf{X})\approx {{\mathbf{b}}}{\varvec{\Phi }}_{\mathbf{X}}\;. \end{aligned}$$

The supplementary information lists the hyper-parameters of ProbCT for each test. It further details the computer hardware we used and the parameters of the optimization processes used during supervised and self-supervised training.

### Cloud tomography products

#### Ground GHI calculation

 An estimate of ${\varvec{\beta }}$ propagates to the estimation of solar ground radiation. We now explain this conversion. Let $I_{\lambda }(\mathbf{X}, {\varvec{\omega }}|{\varvec{\beta }})$ be the radiance field on the ground, for any location $\mathbf{X}$ and direction ${\varvec{\omega }}$, expressed as spectral radiance given ${\varvec{\beta }}$, per wavelength $\lambda$. The interaction of light with cloud droplets is relatively insensitive to $\lambda$ in the visible and near-infrared spectral range. However, $I_{\lambda }(\mathbf{X}, {\varvec{\omega }}|{\varvec{\beta }})$ depends on $\lambda$ due to scattering by air molecules. Let ${\varvec{\chi }}$ be the nadir direction. In the context of solar power generation, radiation power is often quantified^61,62^ by

12 $$\begin{aligned} { \text{GHI}}(\mathbf{X},{{\varvec{\beta }}}) = \int _{\lambda }\int _{{\varvec{\chi }}\cdot {\varvec{\omega }}>0} |{\varvec{\chi }} \cdot {\varvec{\omega }}| I_{\lambda }(\mathbf{X}, {\varvec{\omega }}|{\varvec{\beta }}) d {\varvec{\omega }}d\lambda . \end{aligned}$$

Let ${\varvec{\beta }}^\text{1D}$ be the corresponding estimated cloud extinction coefficient, homogenized horizontally. To assess the error created by 1D cloud approximation, suppose horizontal averaging of cloudy voxels (${\hat{\beta }}>0)$. Then ${\hat{\varvec{\beta }}}^\text{1D}$ varies only in 1D (vertically). The consequent relative error is

13 $$\begin{aligned} \text{GHI}^\text{rel} = \frac{ \text{GHI}[{\hat{\varvec{\beta }}}] - \text{GHI} \left[ {\hat{\varvec{\beta }}}^\text{1D} \right] }{\text{GHI}[{\hat{\varvec{\beta }}}]} \;, \end{aligned}$$

as mapped in Fig. 2D2.

#### Precipitation prospect

 Precipitation is triggered^32^ when the droplet effective radius surpasses a critical value. At location $\mathbf{X}$, the droplet effective radius is $r^\text{e}_\mathbf{X}$. Let $\rho _w \approx 10^6{[\mathrm g/\text{m}^3]}$ be the density of liquid water. The liquid water content (LWC) and these variables are related^12,63^ by

14 $$\begin{aligned} r_\textbf{X}^\text{e}(\beta )=\frac{3{\mathscr {Q}}^\text{eff}}{4\rho _w}\frac{\text{LWC}(\mathbf{X)}}{\beta (\mathbf{X})}\;, \end{aligned}$$

where ${\mathscr {Q}}^\text{eff}$ is the scattering efficiency of droplets, which is $\sim 2$ for visible light. In the core^34^ of a cloud $\text{LWC}(\mathbf{X})\approx \mathrm{LWC^{ad}}({Z})$. The function $\mathrm{LWC^{ad}}({Z})$ is computed^34^, given the cloud base altitude and the vertical temperature profile of the scene. These two parameters are obtained without requiring scattering CT: the cloud base is assessed by space-carving using the multi-view image data^24^. The temperature profile is sampled globally by various meteorological instruments^46^. Ref.^34^ uses typical environmental conditions over the Atlantic near Barbados, plotted in the supplementary information. Overall, $r^\text{e}_\mathbf{X}$ at the cloud core can be approximated by substituting $\text{LWC}(\mathbf{X})$ by $\mathrm{LWC^{ad}}({Z})$ in Eq. (14). We associate a voxel to the cloud core if it is at least 100m away, horizontally, from the cloud edge. Let $\mathbf{X}\in {{\mathscr {Z}}}$ be the set of voxels at altitude *Z* in the cloud core domain. Then, we set $r^\text{e}$ per *Z* using

15 $$\begin{aligned} r^\text{e}({\varvec{\beta }})= \frac{1}{|{{\mathscr {Z}}}|} \sum _{\mathbf{X}\in {{\mathscr {Z}}}} r_\mathbf{X}^\text{e}({{\beta }}_\mathbf{X})\;. \end{aligned}$$

#### Adiabatic fraction

 The 3D *adiabatic fraction* field is the ratio between the estimated $\text{LWC}(\mathbf{X})$ and the theoretical precomputed vertical profile $\mathrm{LWC^{ad}}(Z)$. The estimated ${{\hat{\beta }}}(\mathbf{X})$ is converted to $\text{LWC}(\mathbf{X})$ by Eq. (14), where the 3D fields $r_\mathbf{X}^\text{e}$ is derived using^64^.

## Supplementary Information

Supplementary Information.

## Data Availability

All ProbCT-trained models, the datasets needed to train and evaluate ProbCT, and supplementary data to reproduce the products presented in this paper are publicly and freely available online on the Zenodo platform in https://zenodo.org/records/14796353. The datasets include the training and test sets. These consist of 3D cloud scenes, their corresponding multi-view images, and NASA’s AirMSPI images.

## Code availability

The ProbCT code is freely available at the GitHub (https://github.com/ronenroi/LearnedCloudCT) and Zenodo (https://doi.org/10.5281/zenodo.10579575) repository platforms. These consist of the code to train and validate the model and differentiable rendering codes (for generating cloud images and self-supervised training). The repositories also include use cases and codes that create all downstream products’ results that appear in this manuscript and the supplementary. The SAM code for generating cloud fields is available on the website http://rossby.msrc.sunysb.edu/SAM.html.
