# Supplementary material for: 3D volumetric tomography of clouds using machine learning for climate analysis
Source: Sci Rep. 2025 Mar 10;15:8270. doi: 10.1038/s41598-025-90169-y (PMC11894087; doi:10.1038/s41598-025-90169-y)
Supplement: Supplementary file 1 — Supplementary Information. [file 41598_2025_90169_MOESM1_ESM.pdf]

Supplementary Information
3D volumetric tomography of clouds using machine learning for climate analysis

Roi Ronen[1], Ilan Koren[2], Aviad Levis[3,4], Eshkol Eytan[5,6], Vadim Holodovsky[1], and Yoav Y. Schechner[1,*]

[1]Viterbi Faculty of Electrical & Computer Engineering, Technion - Israel Institute of Technology, Technion City, Haifa, 3200003, Israel
[2]Department of Earth & Planetary Sciences, Weizmann Institute of Science, Herzl St 234, Rehovot, 7610001, Israel
[3]Department of Computer Science, University of Toronto, M5S 2E4, Toronto, Canada
[4]David A. Dunlap Department of Astronomy & Astrophysics, University of Toronto, Toronto, M5S 3H4, Canada
[5]Chemical Sciences Laboratory, National Oceanic and Atmospheric Administration, 325 Broadway, Boulder, 80305, CO, USA
[6]Cooperative Institute for Research in Environmental Sciences, University of Colorado Boulder, 1665 Central Campus Mall, Boulder, 80309, CO, USA
[*]Corresponding author: Yoav Y. Schechner, email address yoav@ee.technion.ac.il

ABSTRACT

This is supplementary material for the main manuscript titled *3D volumetric tomography of clouds using machine learning for climate analysis*.

1 ProbCT Model

1.1 Architecture

In this section, we describe the architecture of the ProbCT model, presented in Fig. 1[A] herein. To ensure the self-contained nature of this supplementary information, we may provide descriptions of details already presented in the main manuscript. ProbCT inputs comprise: image data denoted $\mathbf{y}$, acquired from N^{cam} viewpoints, each indexed c; corresponding 3D camera locations $\{\mathbf{X}_c\}_{c=1}^{N^{\text{cam}}}$; and the 3D coordinates $\mathbf{X}$ of a queried atmospheric domain.

The ProbCT architecture is based on an encoder and a decoder. Here, the encoder *increases* the representation dimension. The encoder is controlled by learned parameters $\Theta^{\text{enc}} = [\Theta^{\text{cam}}, \Theta^{\text{domain}}, \Theta^{\text{image}}]$, detailed below. Per $\mathbf{X}$, the encoder outputs a vector $\mathbf{u}(\mathbf{X}|\Theta^{\text{enc}})$, whose dimensions are much larger than the combined dimensions of voxel and camera poses and the number of image pixels that relate to $\mathbf{X}$. A decoder $\mathscr{D}$ then acts on $\mathbf{u}$, *decreasing* dimensions down to a short, discrete representation of the function $\hat{P}_{\mathbf{X}}(\beta|\mathbf{y})$ at $\mathbf{X}$. The decoder is controlled by learned parameters Θ^{dec}. Overall, the vector of system parameters is

$$\Theta = [\Theta^{\text{enc}}, \Theta^{\text{dec}}] \,. \tag{1}$$

ProbCT thus preforms

$$\hat{P}_{\mathbf{X}}(\beta|\mathbf{y}, \Theta) = \mathscr{D}\left[\mathbf{u}(\mathbf{X}|\Theta^{\text{enc}}), \Theta^{\text{dec}}\right] \,. \tag{2}$$

The encoder includes parallel, independent parts. One part encodes the location $\mathbf{X}_c$ of viewpoint c, yielding an encoded vector $\mathbf{g}^{\text{cam}}(\mathbf{X}_c)$. This encoder part is a deep neural network (DNN) having four fully connected ReLU layers, each layer having 64 neurons (Fig. 1[C]). The neuron weights constitute Θ^{cam}. Using the same Θ^{cam}, this encoder part is applied to all N^{cam} locations in parallel. A similar DNN structure whose neuron weights constitute Θ^{domain} encodes any location $\mathbf{X}$ of a queried voxel. This encoder part yields a vector $\mathbf{g}^{\text{domain}}(\mathbf{X})$.

The image content of relevance to $\mathbf{X}$ is encoded by the following steps, leading to a feature vector $\mathbf{v}(\mathbf{X})$:
`(1)` *Extract a map of image features* across a wide field of view, irrespective of the 3D volumetric element $\mathbf{X}$, using a convolutional DNN, described below. The neuron weights constitute Θ^{image}. The same DNN, using the same Θ^{image}, operates in parallel on all N^{cam} images. To implement this architecture for fast run-time, the N^{cam} images are set in the batch dimension

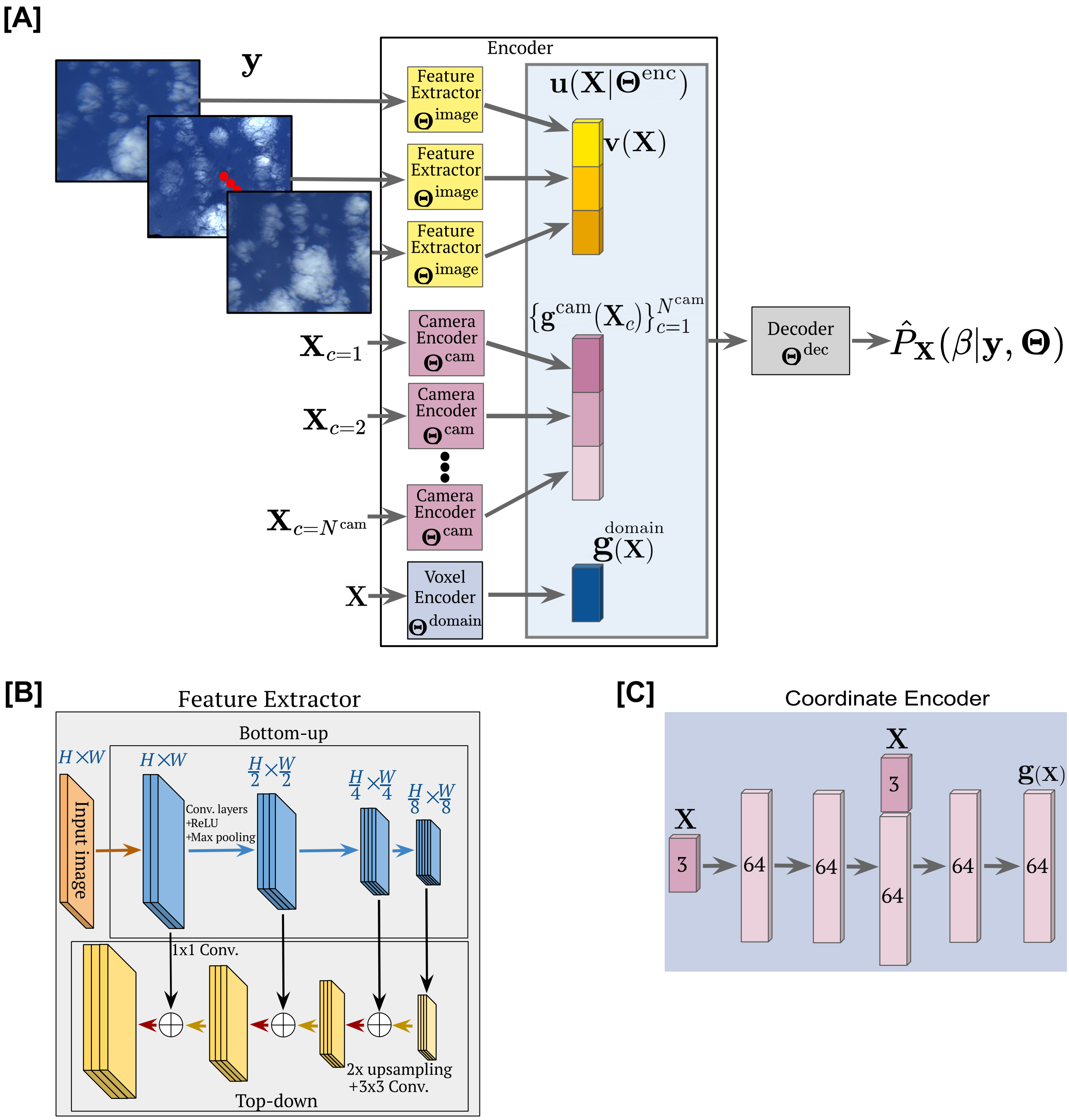

Figure 1. [A] The ProbCT architecture. A 3D scene is observed from N^{cam} viewpoints, yielding multi-image data $\mathbf{y}$. All images are processed by the same feature extractor, yielding corresponding image feature maps. Per location $\mathbf{X}$, the feature maps are sampled at spatial coordinates which correspond to geometric projections of $\mathbf{X}$. This sampling leads to a vector $\mathbf{v}(\mathbf{X})$ of features from all images. 3D coordinates of the location $\mathbf{X}$ and viewpoint locations $\{\mathbf{X}_c\}_{c=1}^{N^{\text{cam}}}$ are processed using coordinate encoders, resulting respectively in geometric feature vectors $\mathbf{g}^{\text{domain}}(\mathbf{X})$ and $\{\mathbf{g}^{\text{cam}}(\mathbf{X}_c)\}_{c=1}^{N^{\text{cam}}}$. These vectors are passed to a decoder that infers the posterior probability distribution of the extinction coefficient $P_{\mathbf{X}}(\beta|\mathbf{y},\Theta)$. [B] A *feature pyramid network* extracts image features. In the bottom-up pathway (blue arrays), each pyramid level reduces resolution by a factor of two in each spatial axis, starting from full resolution at the base level. This pathway utilizes ResNet50[1]. The top-down path consists of four levels (yellow arrays), with lateral connections merging bottom-up and top-down layers of the same spatial size. [C] A 3D coordinate $\mathbf{X}$ is embedded in a 64-dimensional feature vector using fully connected layers with ReLU activation.

of the DNN input.
(2) *Query* $\mathbf{X}$. The location $\mathbf{X}$ is projected to each camera c. This yields a set of *continuous-valued* image locations $\{\mathbf{x}_c\}_{\forall c}$, which correspond to $\mathbf{X}$. (3) *Sample image features* per $\mathbf{x}_c$. The image feature map of step (1) is on a discrete (integer-valued) pixel grid. On the other hand, a projected location $\mathbf{x}_c$ by step (2) is continuous valued, hence generally at intermediate locations between image pixels. Thus, each image feature map is linearly interpolated and re-sampled at $\mathbf{x}_c$.
(4) *Concatenate* corresponding features $\forall c$ to a single vector $\mathbf{v}(\mathbf{X})$.

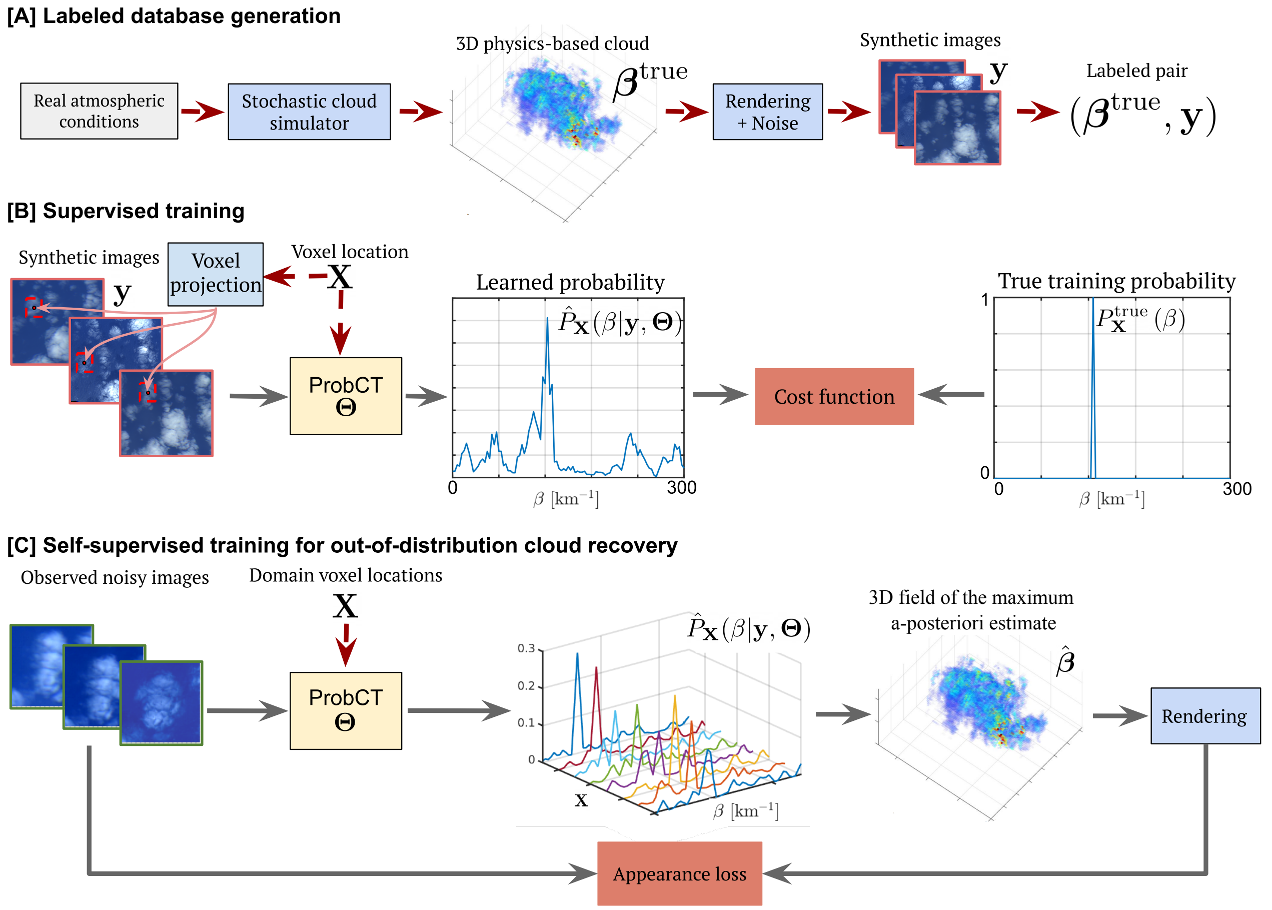

Figure 2. [A] A physics-based cloud simulator generates a random 3D cloud fields β^{true}. The scene is then physically rendered to yield corresponding multi-view images $\mathbf{y}$. [B] Supervised volumetric training of ProbCT (tuning the DNN parameters, Θ). ProbCT trains on pairs of labeled data $(\beta^{\text{true}}, \mathbf{y})$ to estimate a posterior probability distribution, $\hat{P}_{\mathbf{X}}(\beta|\mathbf{y},\Theta)$, per 3D location $\mathbf{X}$. This probability is compared to the true probability at this location, $P_{\mathbf{X}}^{\text{true}}(\beta)$. [C] Self-supervised training improves inference of out-of-distribution clouds. Given observed cloud images, ProbCT estimates $\hat{P}_{\mathbf{X}}(\beta|\mathbf{y},\Theta)$, yielding a maximum a-posteriori estimate $\hat{\beta}$. Using $\hat{\beta}$ in a radiative transfer solver yields re-projected rendered images. These images are compared to the observed images to update Θ.

A map of image features is derived by an off-the-shelf feature pyramid network (FPN)[2], illustrated in Fig. 1[B] herein. A pyramid suits the multi-scale nature of clouds.

The decoder $\mathscr{D}$ has nine fully connected ReLU layers. The final layer of the ProbCT decoder *outputs a vector* of length Q, which is a discrete representation of $\hat{P}_{\mathbf{X}}(\beta|\mathbf{y},\Theta)$. It corresponds to quantized values $\beta(q) = q\Delta\beta$, where $q \in [0,\ldots,Q-1]$ and $\Delta\beta$ is the quantization step of the extinction coefficient. The parameters $Q, \Delta\beta$ were set to cover the entire range of β in the training set that is, $Q\Delta\beta \geq \max\beta$.

1.2 Training

A set of N labeled pairs $\{(\beta_n^{\text{true}}, \mathbf{y}_n)\}_{n=1}^{N}$ are generated for supervised training of Θ via cloud simulators, illustrated in Fig. 2[A] herein. During supervised training (Fig. 2[B] herein), the ProbCT model is exposed to four types of variables: • A large variety of objects (clouds). Thus, the model implicitly learns priors that express what is more probable or less probable to exist in

a voxel of a cloud in the context of other voxels. Hence, the model learns priors on the randomness of nature. • Multi-view images corresponding to each scene. Thus, the model learns to relate objects to images without solving or inverting the radiative transfer (RT) equations. • Random samples of image noise, according to a physical noise model. Thus, the ProbCT model implicitly learns the uncertainty of object recovery relating to sensing noise. • Perturbations to the imaging geometry. Thus, ProbCT learns to generalize CT in variable projections.

There is a disadvantage to supervised training relying on generated clouds. These clouds are based on types (see section 3 herein) emerging from pre-set conditions. Nature tends to be more complicated than anticipated. Often, clouds observed in the wild may deviate from these types (thus the trained distribution). To help ProbCT handle out-of-distribution scenes, supervised training is augmented by *self-supervised* training (see Fig. 2[C] herein), relying on M unlabeled scenes. For them, we only have acquired image data $\{\mathbf{y}_m\}_{m=1}^{M}$, but no corresponding volumetric data. Such data partly correspond to out-of-distribution clouds. While β_m^{true} is unknown, we know the forward model (see section 4 herein) that converts an arbitrary β_m field to rendered multi-view images. The rendered images should have a good *appearance match* to the real acquired data $\mathbf{y}_m$. Hence, Θ can be tuned to optimize an appearance match measure. Such self-supervised learning uses only the physical forward model of RT; hence, it is not sensitive to priors of cloud structure.

We now detail supervised training. For labeled data, the true probability distribution at $\mathbf{X}$ is discretized and represented by a vector, whose q^{th} element is

$$P_{\mathbf{X}}^{\text{true}}(q\Delta\beta) = \begin{cases} 1 & \text{if} \quad q = \lfloor \beta^{\text{true}}(\mathbf{X})/\Delta\beta \rfloor \\ 0 & \text{otherwise} \end{cases} . \tag{3}$$

On the other hand, ProbCT infers a corresponding vector $\hat{P}_{\mathbf{X}}(q\Delta\beta|\mathbf{y},\Theta)$. Training seeks to minimize the distance between these discrete probability distributions. Distance between probability distributions is measured by the Kullback-Leibler (KL) divergence[3]. The cross-entropy of the distributions is

$$\text{CE}_{\mathbf{X}}(\mathbf{y},\Theta) = \text{CE}\{P_{\mathbf{X}}^{\text{true}}(\beta), \hat{P}_{\mathbf{X}}(\beta|\mathbf{y},\Theta)\} \;\; = -\sum_q \left[P_{\mathbf{X}}^{\text{true}}(q\Delta\beta)\log \hat{P}_{\mathbf{X}}(q\Delta\beta|\mathbf{y},\Theta)\right] \;\; = -\log \hat{P}_{\mathbf{X}}\left(\left\lfloor \frac{\beta^{\text{true}}(\mathbf{X})}{\Delta\beta} \right\rfloor \Delta\beta \middle| \mathbf{y},\Theta \right). \tag{4}$$

The last expression in Eq. (4) herein is due to Eq. (3) herein. The entropy[4] of the true distribution at $\mathbf{X}$ is

$$H_{\mathbf{X}}^{\text{true}} = H\{P_{\mathbf{X}}^{\text{true}}(\beta)\} = 0 \,, \tag{5}$$

due to Eq. (3) herein, independently of Θ. The KL divergence is

$$\text{KL}\{P_{\mathbf{X}}^{\text{true}}(\beta), P_{\mathbf{X}}(\beta|\mathbf{y},\Theta)\} = H_{\mathbf{X}}^{\text{true}} + \text{CE}_{\mathbf{X}}(\mathbf{y},\Theta) \,. \tag{6}$$

Based on Eqs. (5,6) herein, the CE criterion (Eq. 4 herein) is the key for optimization.

For self-supervised training, all operations required are differentiable, except an argmax operator for finding the maximum a-posteriori (MAP) estimate. As explained in the main manuscript, we approximate this operator using a differential `Smoothmax` (Boltzmann) operator[5]. Define a distribution

$$\Phi_{\mathbf{X}}(q) = \frac{\left[\hat{P}_{\mathbf{X}}(q\Delta\beta|\mathbf{y},\Theta)\right]^{\alpha}}{\sum_{q'}\left[\hat{P}_{\mathbf{X}}(q'\Delta\beta|\mathbf{y},\Theta)\right]^{\alpha}} \,, \tag{7}$$

where α is a parameter. Fig. 3 herein visualizes $\Phi_{\mathbf{X}}$ for an example posterior probability function $\hat{P}_{\mathbf{X}}(q\Delta\beta|\mathbf{y},\Theta)$.

2 Implementation Details

Supervised training is done by $\approx 100,000$ iterations of stochastic gradient descent via an Adam optimizer. An iteration uses 1000 randomly sampled query voxels. Supervised and self-supervised training use learning rates of 5e-5 and 1e-5, respectively, with a weight decay of 1e-5 and ran on a single NVIDIA GeForce RTX 3090 GPU. During self-supervised training, rendering $\mathscr{F}$ assumes a fixed default cloud phase function and albedo, by setting (as in[6]) the droplet effective radius to $10\mu m$ and effective variance of 0.1. For the results in Fig. 2 of the main manuscript, self-supervised training used $M = 500$ cloud scenes, $Q = 301, \Delta\beta = 1\,\text{km}^{-1}$, and $w_{\mathbf{X},n}^{\text{cloud}} = 0.01$ for non-cloudy voxels. The same values of Q and $w_{\mathbf{X},n}^{\text{cloud}}$ were used for the results in Fig. 3 of the main manuscript.

Results in Fig. 4 of the main manuscript use real AirMSPI data. AirMSPI imaging is done sequentially during ≈ 10 minutes along a flight path. During this time, clouds drift due to wind. Thus, as a pre-process, we follow[6–8] to assess and compensate for global drift between images. Self-supervised learning uses $M = 3$ clouds of similar characteristics in the field of view. After training, we inferred a fourth volumetric domain. Additionally, we set in this experiment the following hyperparameters: $Q = 101, \Delta\beta = 0.5\,\text{km}^{-1}$ and $w_{\mathbf{X},n}^{\text{cloud}} = 0.1$ for non-cloudy voxels. In all tests, we use $\alpha = 10$ in Eq. (7) herein.

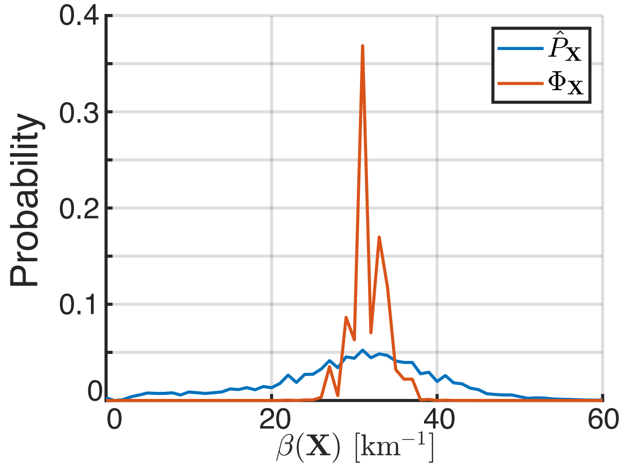

Figure 3. Visualization of the differential `Smoothmax` (Boltzmann) operator[5] in Eq. (7) herein. Self-supervised training uses this form.

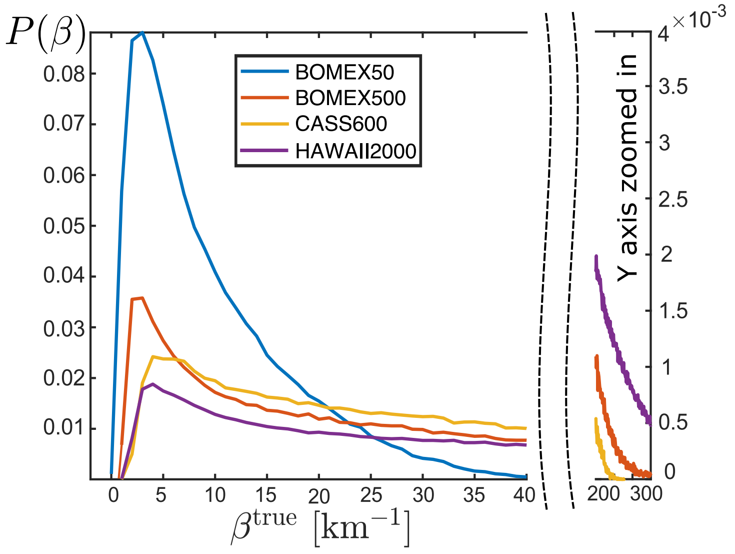

Figure 4. Statistics of four classes of simulated cumulus clouds based on empirical environmental boundary conditions.

3 Synthetic 3D Clouds

Validated dynamical system simulators form synthetic, physics-based clouds. The simulators rely on initial atmospheric conditions, including aerosols. There are well-studied initial atmospheric conditions termed BOMEX, CASS, and HAWAII. We have derived several classes (thus sets) of simulated cloud fields based on them. In this work, we derive datasets BOMEX50 and HAWAII2000 to enable out-of-distribution studies, expanding smaller datasets[9] termed BOMEX500 and CASS600. The suffix number is the aerosol concentration in particles/cm^3. We also use an augmentation using the BOMEX500-Aux dataset: there, for each cloud of the BOMEX500 set, the liquid water content (LWC) is multiplied by 1/10. Some statistics of these classes appear in Fig. 4 herein and Table 1a herein.

Environmental conditions include aerosol concentrations. The aerosol concentration affects clouds in two main ways. First, aerosols affect feedback of cloud dynamics, consequently affecting the morphology of clouds and cloud fields. Second, a higher aerosol concentration leads to a higher concentration of cloud droplets, yet of smaller droplet size. These changes, in turn, affect the internal structure of the β field in the cloud. Moreover, droplet size affects the scattering phase function and single-scattering albedo of the droplets, following Mie theory. These parameters then affect image formation, as described in Sec. 4 herein.

Each class of clouds has a different statistical distribution. The cloud sets have different spatial textures spanning different domain and voxel sizes. Per class, a simulation results in a labeled ground-truth volumetric scene. A spatially varying field of

Dataset	#Train	#Test	Voxel size [m]	Grid size	Camera	Pixel footprint
BOMEX50	1660	203	$50 \times 50 \times 40$	$32 \times 32 \times 64$	perspective	20[m]
BOMEX500	6001	566	$50 \times 50 \times 40$	$32 \times 32 \times 32$	perspective	20[m]
CASS600	10908	1000	$50 \times 50 \times 40$	$64 \times 64 \times 32$	perspective	20[m]
HAWAII2000	1227	722	$50 \times 50 \times 20$	$32 \times 32 \times 64$	perspective	20[m]
BOMEX500-Aux	4418	$\times$	$50 \times 50 \times 40$	$32 \times 32 \times 32$	pushbroom	10[m]

(a)

Train \Test	BOMEX50	BOMEX500	CASS600	HAWAII2000
BOMEX50	0.33±0.11	$\times$	$\times$	$\times$
BOMEX500	0.49±0.10	0.33±0.13	0.36±0.14	0.52±0.20
CASS600	$\times$	0.65±0.13	0.22±0.06	$\times$
HAWAII2000	$\times$	$\times$	$\times$	0.44±0.17
Physics-based[6]	0.51±0.07	0.97±0.24	0.76±0.38	0.82±0.27

(b)

Table 1. (a) Specifications of simulated cloud datasets. Each has a different number of training and testing examples, as well as voxel size resolution and domain size. This diversity requires flexibility in the analysis system. The BOMEX500 and BOMEX500-Aux sets include perturbations to the imaging geometry. (b) Results of cloud volumetric recovery, measured by the mean and standard deviation of ε (Eq. 12 herein). Each row represents the training class of clouds from which examples are drawn for supervised training. Columns refer to the test class from which clouds are drawn. The main diagonal summarizes in-distribution inference errors. Off-diagonals apply to out-of-distribution inference. ProbCT outperforms an existing physics-based solver[6] for both in-distribution and out-of-distribution tests across all datasets. Moreover, ProbCT inference is about $\times 1000$ faster than the physics-based solver.

the optical extinction coefficient characterizes a scene. A vector β^{true} represents this coefficient in a grid of voxels. Each scene can be converted to corresponding image data (denoted $\mathbf{y}$) at any optical wavelength and viewpoint poses using RT. The cloud scenes and the corresponding images constitute labeled databases.

We focus on the CloudCT[10,11] formation: 10 nano-satellites, having 100km between nearest neighboring, perspective viewpoints, orbiting 500km high. We use a viewing geometry similar to that. The solar zenith angle is 25°. We use a spectral band around 670 nm and random image noise, whose specifications are typical to the CloudCT payload (see Methods section). Rendered images have 116×116 pixels, with 20 m/pixel at nadir.

Additionally, we use rendered images corresponding to NASA's AirMSPI instrument. It takes nine pushbroom multi-angular images in a $\pm 67^\circ$ angular span along the flight path at 20 km altitude, with 10 m resolution at nadir, around wavelength 660 nm. The rendering uses the synthetic cloud scenes from the BOMEX500-Aux dataset and five different AirMSPI flight experiments with random viewpoint perturbations. The rendered AirMSPI training images use random radiometric noise, having the specifications[12] of this sensor. The ground surface is Lambertian for all rendered images with an albedo 0.05.

3.1 Probing a single voxel posterior

In the case study illustrated in Fig. 3 of the main manuscript, the dataset is made of clouds in which one voxel has a random extinction coefficient β, sampled from a bimodal probability distribution. The probability distribution is

$$P^{\text{true}}(\beta) = \begin{cases} \beta^{\text{low}} & \text{with probability } 3/4 \\ \beta^{\text{high}} & \text{otherwise} \end{cases}, \tag{8}$$

where $\beta^{\text{low}}, \beta^{\text{high}}$ are normally distributed with expectations $42\,\text{km}^{-1}$ and $75\,\text{km}^{-1}$, respectively, and have the same standard deviation of $5\,\text{km}^{-1}$.

4 3D Radiative Transfer

Rendering ground-truth images uses 3D RT of incoherent light in a heterogeneous medium, as illustrated in Fig. 5 herein. A 3D location is denoted $\mathbf{X}$. Besides cloud droplets, air molecules affect RT. Throughout the paper, we model the molecular

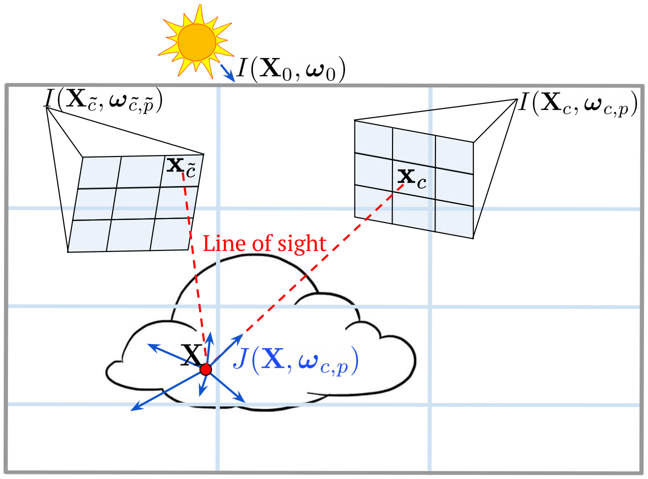

Figure 5. At boundary point $\mathbf{X}_0$, known radiance $I(\mathbf{X}_0,\omega_0)$ is incident in direction ω_0. Radiance scatters multiple times in the domain. The 3D functions J (Eq. 11 herein) and the extinction coefficient β define the radiance field I by Eq. (10) herein. Pixel p of camera c corresponds line of sight to direction $\omega_{c,p}$. This pixel samples the radiance $I(\mathbf{X}_c,\omega_{c,p})$.

extinction coefficient $\beta^{\text{air}}(\mathbf{X})$ using a summer mid-latitude vertical distribution[6], at altitudes in the range $[0,20]$km. Atmospheric transmittance between any two points $\mathbf{X}'$, $\mathbf{X}''$ is

$$T(\mathbf{X}',\mathbf{X}'') = \exp\left[-\int_{\mathbf{X}'}^{\mathbf{X}''}\{\beta(\mathbf{X})+\beta^{\text{air}}(\mathbf{X})\}d\mathbf{X}\right]. \tag{9}$$

The medium is also characterized by a single-scattering albedo $\varpi(\mathbf{X})$, and a scattering phase function. The phase function $\mathrm{p}(\mathbf{X},\omega\cdot\omega')$ expresses the relative portion of radiance scattered to the 3D direction unit-vector ω, when radiance is incident at $\mathbf{X}$ in direction ω'. The values of ϖ and p stem from microphysical properties of the mixture[13] of particles in a voxel around $\mathbf{X}$, including air and water droplets.

At boundary point $\mathbf{X}_0$ of the observed domain, the incident radiance $I(\mathbf{X}_0,\omega)$ in direction ω is known. Radiation is then affected by the medium, generally multiple times, by interactions of scattering and absorption. As a result, a radiance field $I(\mathbf{X},\omega)$ encompasses the scene domain in all directions. This process is modeled by coupled and recursive 3D RT equations[14], sometimes referred to as *volume rendering* equations,

$$I(\mathbf{X},\omega) = I(\mathbf{X}_0,\omega)T(\mathbf{X}_0,\mathbf{X}) + \int_{\mathbf{X}_0}^{\mathbf{X}} J(\mathbf{X}',\omega)\left[\beta(\mathbf{X}')+\beta^{\text{air}}(\mathbf{X}')\right]T(\mathbf{X}',\mathbf{X})d\mathbf{X}' , \tag{10}$$

$$J(\mathbf{X},\omega) = \frac{\varpi(\mathbf{X})}{4\pi}\int_{4\pi}\mathrm{p}(\mathbf{X},\omega\cdot\omega')I(\mathbf{X},\omega')d\omega' . \tag{11}$$

The radiance field is projected to N^{cam} observational cameras. Camera c has a 3D center of projection at $\mathbf{X}_c$. In this camera, pixel p corresponds to a line of sight having a particular direction, denoted $\omega_{c,p}$. Projection of the scene to this pixel in this camera amounts to sampling the radiance field at $I(\mathbf{X}_c,\omega_{c,p})$. Overall, the *forward model* $\mathscr{F}(\beta)$ constitutes 3D RT followed by projection to all cameras and consequent sampling to pixels. In this paper, as in[6,7], the scattering particles have known ϖ while $\mathrm{p}(\mathbf{X},\omega\cdot\omega')$ is approximately known. The recursive nature of 3D radiative transfer (Eqs. 9,10,11 herein) makes it impossible to solve in finite time. Hence, approximate solutions are used numerically. This paper uses the SHDOM[15,16] RT solver. However, Monte Carlo methods can also be used.

5 Numerical results

The extinction field $\beta(\mathbf{X})$ can be sampled on a voxel grid to form a vector $\boldsymbol{\beta}$. To quantitatively evaluate the MAP performance, we follow[6,7,9] and use per scene these criteria

$$\varepsilon = \frac{\|\boldsymbol{\beta}^{\text{true}}-\hat{\boldsymbol{\beta}}\|_1}{\|\boldsymbol{\beta}^{\text{true}}\|_1}\ ,\ \delta = \frac{\|\boldsymbol{\beta}^{\text{true}}\|_1-\|\hat{\boldsymbol{\beta}}\|_1}{\|\boldsymbol{\beta}^{\text{true}}\|_1}\ , \tag{12}$$

Table 2. A comparison based on the BOMEX50 dataset. ProbCT can lead to an estimate $\hat{\beta}$ that achieves maximum a-posteriori (MAP) probability or, alternatively, an expected (mean) value. Physics-based[6] recovery time is about $\times 1000$ longer than VIP-CT and ProbCT. We show the mean and standard deviation of the ε, δ criteria over all examples. ProbCT outperforms prior art.

Test	Method	$\varepsilon\% \downarrow$	$\delta\% \rightarrow 0$
ID	VIP-CT[7]	31 ± 10	16 ± 12
	ProbCT (MAP)	30 ± 10	15 ± 12
	ProbCT (mean)	31 ± 11	13 ± 14
OOD	VIP-CT[7]	54 ± 10	12 ± 13
	ProbCT (MAP)	49 ± 10	15 ± 12
	ProbCT (mean)	57 ± 15	-8 ± 24
	Physics-based[6]	51 ± 08	31 ± 12

Table 3. An ablation study of the quantization step $\Delta\beta$ in an ID test. A smaller $\Delta\beta$ requires a model of higher complexity and possibly more data to train sufficiently. A good balance of accuracy vs. complexity is achieved at $\Delta\beta = 1\,\text{km}^{-1}$.

$\Delta\beta\ \text{km}^{-1}$	0.1	0.5	1	2	10
$\varepsilon\%$	34 ± 11	32 ± 12	33 ± 11	37 ± 12	57 ± 14

where $0 \leq \varepsilon$ and $-1 \leq \delta \leq 1$. Ideally, $\varepsilon = 0$ and $\delta = 0$.

Table 1b herein compares ProbCT results for different cloud classes, where each row represents the dataset from which 3D cloud examples are drawn for supervised training. Therefore, the main diagonal in Table 1b herein summarizes in-distribution (ID) inference errors, while off-diagonal results in Table 1b herein apply to out-of-distribution (OOD) inference. For example, the column titled BOMEX50 refers to testing on the BOMEX50 clouds. The results in the first and second rows involve supervised training using BOMEX50 or BOMEX500, respectively, followed by refinement using images (not 3D clouds) that correspond to clouds from the BOMEX50 training set.

Over the test sets, ProbCT outperforms a physics-based method[6], which uses iterative optimization by differential rendering. Moreover, ProbCT requires, on average, less than a second per inferred scene when computation uses a single GPU. In contrast, recovery using physics-based differential rendering[6] requires ~ 1000 seconds.

In Table 2 herein, we detail numerical results for simulations described and plotted in the main manuscript for the BOMEX50 dataset. Furthermore, we conducted ablation studies to evaluate how the quantization step $\Delta\beta$ affects the ε measure. The results are listed in Table 3 herein.

5.1 Interpretable example

In this section, we design an interpretable example to assess what ProbCT learns and infers. The tests in Fig. 3 of the main manuscript and in Fig. 6 herein provide evidence supporting that ProbCT infers $P_{\mathbf{X}}(\beta|\mathbf{y})$, having similarity to a true $P_{\mathbf{X}}^{\text{true}}(\beta|\mathbf{y})$ in limit and intermediate cases.

Consider a spherical object ("spherical cloud") having three concentric parts (Fig. 6[A] herein): A core having an unknown β^{core}; an intermediate shell, whose optical thickness is known and very high; and an outer shell having an unknown β^{outer}. The clouds are observed from space by the geometry of the CloudCT formation. Voxels of the outer shell, mainly those on top, are directly exposed to light and the cameras. Hence, measurements are sensitive to β^{outer}. We thus expect $\hat{\beta}$ in any outer voxel to be both accurate (close to β^{outer}) and having low uncertainty, being rather insensitive to the prior of the probability distribution of β^{outer}, denoted $P^{\text{outer}}(\beta)$.

On the other hand, the middle of the sphere is *veiled* by the optically thick intermediate shell (a veiled core[17,18]). Light undergoes many scattering events until it reaches the core and, afterward, on the way to the cameras. The camera measurements have noise, which overwhelms the core's signal. Hence, the measured signal is oblivious to β^{core}. Therefore, we expect the estimation of $\hat{\beta}$ in core voxels to be random, relying only on the prior probability distribution of β^{core}, denoted $P^{\text{core}}(\beta)$, on which the system had trained.

We generated 550 synthetic spherical clouds for training and 100 for testing. Each has spatially uniform shells with

$$\beta(\mathbf{X}) = \begin{cases} \beta^{\text{core}} & \|\mathbf{X}-\mathbf{O}\|_2 \leq 60\,\text{m} \\ \beta^{\text{inter}} & 60\,\text{m} < \|\mathbf{X}-\mathbf{O}\|_2 \leq 500\,\text{m} \\ \beta^{\text{outer}} & 500\,\text{m} < \|\mathbf{X}-\mathbf{O}\|_2 \leq 600\,\text{m} \\ 0 & \text{otherwise} \end{cases}, \tag{13}$$

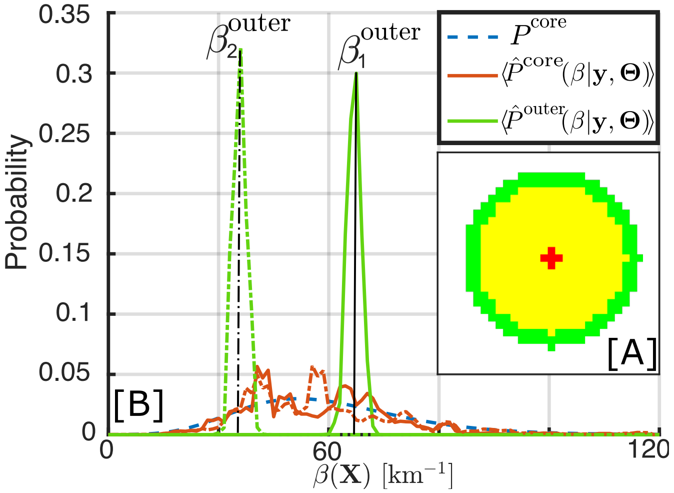

Figure 6. Red, yellow, and green represent the cloud core, intermediate, and outer shells, respectively. [A] A cross-section through the center of the spherical cloud. [B] Plots for two different clouds in the test set, β_1, β_2, marked by solid and dash lines. They show the true (blue) probability distribution of the core, estimated (red) posterior probability distributions of the core, and estimated outer-shell posterior probability distributions (green). The actual sampled outer-shell values are $\beta_1^{\text{outer}}, \beta_2^{\text{outer}}$.

where $\mathbf{O} = (0.8, 0.8, 1.28)$km. In the intermediate shell, $\beta^{\text{inter}} = 190\text{km}^{-1}$ (visibility of ≈ 5m). In each cloud, the random values $\beta^{\text{core}}, \beta^{\text{outer}}$ are drawn independently of each other from a log-normal probability

$$P^{\text{outer}}(\beta) = P^{\text{core}}(\beta) = l(160/\beta)\exp\{-8[\ln(\beta/160)+1]^2]\}\ , \tag{14}$$

where l is a normalization constant and β is in km^{-1}, having expectation $\approx 61\text{km}^{-1}$ and standard deviation $\approx 15\text{km}^{-1}$.

After inference, let us empirically average the inferred probability distribution per shell. For example, let $|\mathbf{X}^{\text{core}}|$ be the number of core voxels. The spatially averaged inferred probability is

$$\langle\hat{P}^{\text{core}}(\beta|\mathbf{y},\Theta)\rangle = \frac{1}{|\mathbf{X}^{\text{core}}|}\sum_{\mathbf{X}^{\text{core}}} \hat{P}^{\text{core}}_{\mathbf{X}}(\beta|\mathbf{y},\Theta)\ . \tag{15}$$

Fig. 6[B] herein plots inferred probability distributions in two clouds. As expected (red lines), $\langle\hat{P}^{\text{core}}(\beta|\mathbf{y},\Theta)\rangle \sim P^{\text{core}}(\beta)$. On the other hand, $\langle\hat{P}^{\text{outer}}(\beta|\mathbf{y},\Theta)\rangle$ is sharply peaked at the correct ground truth β^{outer}, per cloud, with low uncertainty (green lines).

6 Global horizontal irradiance calculations

In this section, we detail the calculation of the global horizontal irradiance (GHI)[19,20], due to solar energy (see Fig. 7 herein). Calculation of irradiance on the ground involves $\{i\}$ solar irradiance at the top of the atmosphere, $\{ii\}$ atmospheric extinction (including by clouds), leading to directly-transmitted solar irradiance, and $\{iii\}$ 3D RT by the atmosphere, yielding diffuse sky irradiance.

Fig. 8 herein shows the experimentally measured solar irradiance[21] at the top of the atmosphere. Using irradiance at the top of the atmosphere and given atmospheric content, RT calculations yield the radiance field $I_\lambda(\mathbf{X},\omega|\beta)$, for any location $\mathbf{X}$ and direction ω, per wavelength λ. The interaction of light with cloud droplets is relatively insensitive to λ in the visible and near-infrared spectral range Λ. Therefore, we omit the dependency of the cloud extinction coefficient β on λ. However, RT depends on λ due to scattering by air molecules.

Let χ denote the nadir direction. For a location on the ground, only light coming from the upper hemisphere[22] is relevant. Accordingly, GHI on the ground is

$$\text{GHI}(\mathbf{X},\beta) = \int_\lambda \int_{\chi\cdot\omega>0} |\chi\cdot\omega| I_\lambda(\mathbf{X},\omega|\beta)\, d\omega\, d\lambda. \tag{16}$$

in units of $[\frac{\text{W}}{\text{m}^2}]$.

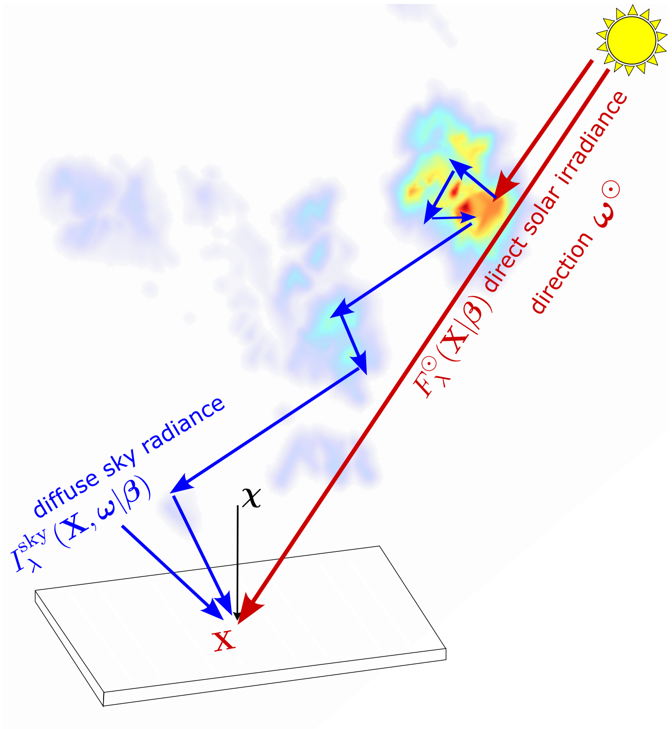

Figure 7. Direct solar irradiance and diffuse sky radiation. Both reach the ground and are influenced by the atmosphere.

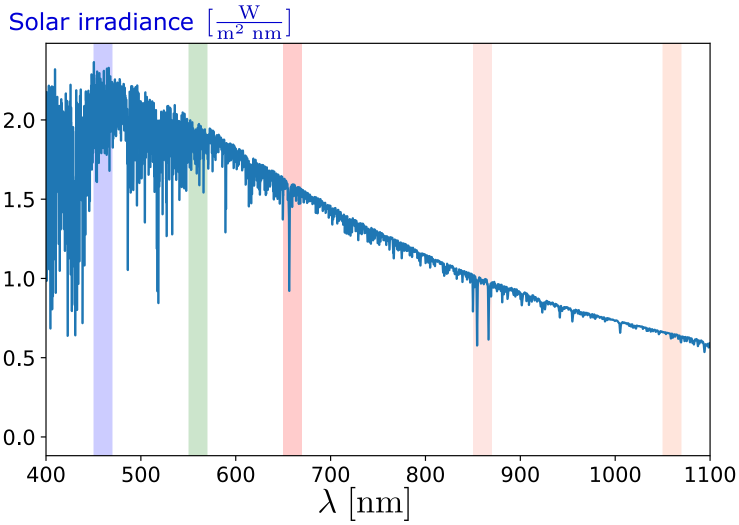

Figure 8. [Blue] Solar irradiance spectrum at the top of the atmosphere. Five wavebands are marked, each with a bandwidth of 20 nm. They sample the spectral functions.

We now provide implementation details. Direct solar irradiance arrives at direction $\boldsymbol{\omega}^{\odot}$, having a corresponding zenith angle $\Psi(\boldsymbol{\omega}^{\odot})$. This irradiance on the ground is denoted $F_\lambda^{\odot}(\mathbf{X}|\beta)$, and accounts both for extinction by the atmosphere and the $|\chi \cdot \boldsymbol{\omega}^{\odot}|$ factor that appears in Eq. (16) herein. Eq. (16) herein can be divided to two components

$$\text{GHI}(\mathbf{X},\beta) = \int_\lambda \left[F_\lambda^{\odot}(\mathbf{X}|\beta) + \int_{\chi\cdot\boldsymbol{\omega}>0} |\chi \cdot \boldsymbol{\omega}| I_\lambda^{\text{sky}}(\mathbf{X},\boldsymbol{\omega}|\beta)\, d\boldsymbol{\omega} \right] d\lambda, \tag{17}$$

where $I_\lambda^{\text{sky}}(\mathbf{X},\boldsymbol{\omega}|\beta)$ is the diffuse sky irradiance. In our implementation, we obtain the fields $I_\lambda^{\text{sky}}(\mathbf{X},\boldsymbol{\omega}|\beta)$ and $F_\lambda^{\odot}(\mathbf{X}|\beta)$ using the AT3D[16] code package. AT3D wraps a spherical harmonic discrete ordinate method (SHDOM) code of RT.

We compute the integral in Eq. (17) herein by sampling Λ at [460,560,660,860,1060]nm, where each waveband is 20nm

wide. The other simulation parameters of the AT3D[16] RT are as follows: $\Psi(\omega^{\odot}) = 25°$; the air model set to a summer mid-latitude vertical distribution[6] at altitudes in the [0,20]km range; the droplet effective radius is 10μm and the effective variance is 0.1. The ground surface is Lambertian with an albedo of 0.05.

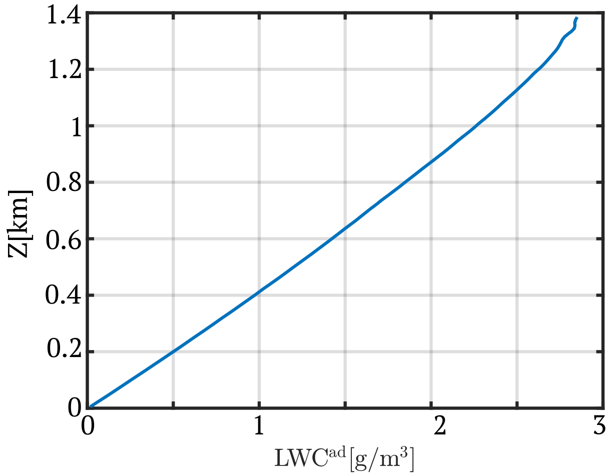

Figure 9. A plot of the cloud liquid water content (LWC) as a function of altitude Z above the cloud base, according to the adiabatic model.

7 Liquid water content

According to the adiabatic model, in the core of a cloud, the liquid water content (LWC) is a function of the altitude Z[23] above the cloud base, that is,

$$\mathrm{LWC}(\mathbf{X}) \approx \mathrm{LWC}^{\mathrm{ad}}(Z) \ . \tag{18}$$

The function $\mathrm{LWC}^{\mathrm{ad}}(Z)$ can be calculated[23]. Such a function is shown in the supplementary Fig. 9 herein.

References

1. He, K., Zhang, X., Ren, S. & Sun, J. Deep residual learning for image recognition. In *Proceedings of the IEEE conference on computer vision and pattern recognition*, 770–778 (2016).
2. Lin, T.-Y. *et al.* Feature pyramid networks for object detection. In *Proceedings IEEE/CVF Conference on Computer Vision and Pattern Recognition*, 2117–2125 (2017).
3. Joyce, J. M. Kullback-Leibler divergence. In *International encyclopedia of statistical science*, 720–722 (Springer, 2011).
4. Shannon, C. E. A mathematical theory of communication. *The Bell system technical journal* **27**, 379–423 (1948).
5. Asadi, K. & Littman, M. L. An alternative softmax operator for reinforcement learning. *Int. Conf. on Mach. Learn.* 243–252 (2017).
6. Levis, A., Schechner, Y. Y., Aides, A. & Davis, A. B. Airborne three-dimensional cloud tomography. In *Proceedings IEEE/CVF International Conference on Computer Vision*, 3379–3387 (2015).
7. Ronen, R., Holodovsky, V. & Schechner, Y. Y. Variable imaging projection cloud scattering tomography. *IEEE Transactions on Pattern Analysis Mach. Intell.* 1–12 (2022).
8. Ronen, R., Schechner, Y. Y. & Eytan, E. 4D cloud scattering tomography. In *Proc. IEEE/CVF International Conference on Computer Vision*, 5520–5529 (2021).
9. Sde-Chen, Y., Schechner, Y. Y., Holodovsky, V. & Eytan, E. 3DeepCT: Learning volumetric scattering tomography of clouds. In *Proceedings IEEE/CVF International Conference on Computer Vision*, 5671–5682 (2021).
10. Schilling, K., Schechner, Y. Y. & Koren, I. CloudCT - computed tomography of clouds by a small satellite formation. In *Proceedings IAA Symposium on Small Satellites for Earth Observation* (2019).

11. Tzabari, M. *et al.* CloudCT 3D volumetric tomography: Considerations for imager preference, comparing visible light, short-wave infrared, and polarized imagers. In *Polarization Science and Remote Sensing X*, vol. 11833, 19–26 (SPIE, 2021).

12. NASA. AirMSPI version 5 ellipsoid-projected georegistered radiance product acquired during the NASA PODEX flight campaign january-february 2013 (2013).

13. Levis, A., Schechner, Y. Y. & Davis, A. B. Multiple-scattering microphysics tomography. In *Proceedings of the IEEE Conference on Computer Vision and Pattern Recognition*, 6740–6749 (2017).

14. Chandrasekhar, S. *Radiative Transfer* (Courier Corporation, 1960).

15. Levis, A., Loveridge, J. & Aides, A. *PySHDOM. 2020. Available online*. https://github.com/aviadlevis/pyshdom.

16. Loveridge, J., Levis, A., Aides, A., Forster, L. & Holodovsky, V. Atmospheric tomography with 3D radiative transfer (2022). https://github.com/CloudTomography/AT3D.

17. Forster, L., Davis, A. B., Diner, D. J. & Mayer, B. Toward cloud tomography from space using MISR and MODIS: Locating the "veiled core" in opaque convective clouds. *J. Atmospheric Sci.* **78**, 155–166 (2021).

18. Loveridge, J. *et al.* Retrieving 3D distributions of atmospheric particles using atmospheric tomography with 3D radiative transfer–part 1: Model description and Jacobian calculation. *Atmospheric Meas. Tech.* **16**, 1803–1847 (2023).

19. Duffie, J. A. & Beckman, W. A. *Solar engineering of thermal processes* (John Wiley & Sons, 2013).

20. Xie, Y., Sengupta, M., Habte, A. & Andreas, A. The "Fresnel equations" for diffuse radiation on inclined photovoltaic surfaces (FEDIS). *Renew. Sustain. Energy Rev.* **161**, 112362 (2022).

21. Wehrli, C. Extraterrestrial solar spectrum, publication no. 615. *PMO/WRC, Davos Dorf, Switz.* (1985).

22. Marshak, A. & Davis, A. *3D radiative transfer in cloudy atmospheres* (Springer, 2005).

23. Eytan, E., Koren, I., Altaratz, O., Pinsky, M. & Khain, A. Revisiting adiabatic fraction estimations in cumulus clouds: high-resolution simulations with a passive tracer. *Atmospheric Chem. Phys.* **21**, 16203–16217 (2021).
